# Hierarchical Swin Transformer Ensemble with Explainable AI for Robust and Decentralized Breast Cancer Diagnosis

**DOI:** 10.3390/bioengineering12060651

**Published:** 2025-06-13

**Authors:** Md. Redwan Ahmed, Hamdadur Rahman, Zishad Hossain Limon, Md Ismail Hossain Siddiqui, Mahbub Alam Khan, Al Shahriar Uddin Khondakar Pranta, Rezaul Haque, S M Masfequier Rahman Swapno, Young-Im Cho, Mohamed S. Abdallah

**Affiliations:** 1 Department of Computer Science and Engineering, East West University, Dhaka 1212, Bangladeshrezaulh603@gmail.com (R.H.); 2 Department of Management Information System, International American University, 3440 Wilshire Blvd. STE 1000, Los Angeles, CA 90010, USA; 3 Department of Computer Science, Westcliff University, Irvine, CA 92614, USA; 4 Department of Engineering/Industrial Management, Westcliff University, Irvine, CA 92614, USA; 5 Department of Management Information System, Pacific State University, 3424 Wilshire Blvd., 12th Floor, Los Angeles, CA 90010, USA; 6 Department of Computer Science, Wright State University, 3640 Colonel Glenn Hwy, Dayton, OH 45435, USA; 7 Department of Computer Science and Engineering, Bangladesh University of Business and Technology, Dhaka 1216, Bangladesh; masfequier.cse.bubt@gmail.com; 8 Department of Computer Engineering, Gachon University, Seongnam 13415, Republic of Korea; 9 Informatics Department, Electronics Research Institute (ERI), Cairo 11843, Egypt

**Keywords:** federated learning, breast cancer, vision transformers, ensemble learning, privacy-preserving, clinical decision support

## Abstract

Early and accurate detection of breast cancer is essential for reducing mortality rates and improving clinical outcomes. However, deep learning (DL) models used in healthcare face significant challenges, including concerns about data privacy, domain-specific overfitting, and limited interpretability. To address these issues, we propose BreastSwinFedNetX, a federated learning (FL)-enabled ensemble system that combines four hierarchical variants of the Swin Transformer (Tiny, Small, Base, and Large) with a Random Forest (RF) meta-learner. By utilizing FL, our approach ensures collaborative model training across decentralized and institution-specific datasets while preserving data locality and preventing raw patient data exposure. The model exhibits strong generalization and performs exceptionally well across five benchmark datasets—BreakHis, BUSI, INbreast, CBIS-DDSM, and a Combined dataset—achieving an F1 score of 99.34% on BreakHis, a PR AUC of 98.89% on INbreast, and a Matthews Correlation Coefficient (MCC) of 99.61% on the Combined dataset. To enhance transparency and clinical adoption, we incorporate explainable AI (XAI) through Grad-CAM, which highlights class-discriminative features. Additionally, we deploy the model in a real-time web application that supports uncertainty-aware predictions and clinician interaction and ensures compliance with GDPR and HIPAA through secure federated deployment. Extensive ablation studies and paired statistical analyses further confirm the significance and robustness of each architectural component. By integrating transformer-based architectures, secure collaborative training, and explainable outputs, BreastSwinFedNetX provides a scalable and trustworthy AI solution for real-world breast cancer diagnostics.

## 1. Introduction

Breast cancer is a substantial global health issue and is the most commonly diagnosed cancer among women worldwide [1]. It also ranks as a leading cause of cancer-related deaths. According to the World Health Organization (WHO), approximately 2.3 million new cases of breast cancer and 685,000 related deaths were reported in 2020, accounting for nearly 12% of all newly diagnosed cancers globally [2,3]. The lifetime risk of a woman developing breast cancer is estimated to be 1 in 8, and the prognosis is heavily influenced by early detection and access to effective treatment options [4]. Conventional diagnostic methods, such as mammography, ultrasound, and histopathological examination, rely on manual interpretation by clinicians. This reliance introduces subjectivity and variability between different observers and can lead to delays in diagnosis [5,6]. Moreover, imaging modalities such as histopathology, mammography, and ultrasound introduce additional diagnostic variability due to differences in acquisition protocols and tissue characteristics.

To address these challenges, DL-based computer-aided diagnostic (CAD) systems have demonstrated notable promise in automating and improving breast cancer classification. However, centralized DL models require large amounts of labeled data collected from multiple institutions, which raises serious concerns regarding data privacy, security, and compliance with regulations such as HIPAA and GDPR [7,8]. Furthermore, models trained on datasets from a single source often struggle to generalize effectively to external data due to differences in domain and variations in image acquisition protocols. This can lead to significant performance drops—sometimes exceeding 20%—when tested on unseen datasets [9]. Another common challenge in breast cancer diagnosis is data imbalance, where certain minority classes, such as specific subtypes of malignant tumors, are underrepresented. This imbalance results in biased model learning, higher false-negative rates, and reduced clinical reliability in detecting rare but critical conditions. There is an urgent need for improved automated systems capable of cross-institutional generalization and equitable classification performance, particularly for rare malignant subtypes.

FL has emerged as a promising approach for preserving privacy and allows for collaborative training of DL models across decentralized institutions without exposing raw data [10,11]. By keeping data localized, FL ensures compliance with regulatory requirements and enhances model robustness by exposing it to diverse data distributions. However, Convolution Neural Network (CNN)-based FL solutions often fall short in modeling long-range dependencies and adapting to non-IID clinical data, limiting their utility for high-resolution medical imaging tasks [12,13]. Furthermore, these approaches struggle to effectively model multi-scale contextual patterns that are critical for analyzing high-resolution breast cancer images [14,15]. To address these limitations, Vision Transformers (ViTs) have recently gained prominence for their ability to model global spatial relationships [16]. Among them, the Swin Transformer introduces a hierarchical, window-shifted self-attention mechanism that balances accuracy and computational efficiency, making it particularly effective in capturing both fine-grained and contextual features across multi-resolution breast imaging datasets [17,18]. However, standalone transformer models can still be prone to overfitting, especially in situations with limited or imbalanced data [19]. To improve generalization and stability, ensemble learning strategies that combine multiple variants of the Swin Transformer can be beneficial, particularly when trained on diverse datasets.

A key factor that limits the adoption of AI models in clinical workflows is their lack of transparency. Most DL models operate as “black boxes”, making it difficult for healthcare professionals to understand or trust the reasoning behind their predictions. To mitigate this, techniques such as Gradient-weighted Class Activation Mapping (Grad-CAM) offer interpretable visual feedback, allowing clinicians to assess the model’s focus regions in relation to known pathology. This approach helps bridge the gap between automated inference and human interpretation [20]. Studies show that only 15% of clinicians express full confidence in AI systems that lack interpretability [21]. Explainability not only achieves clinical trust but also improves safety, regulatory accountability, and ethical deployment in high-stakes environments such as oncology. Another vital yet often overlooked aspect of real-world deployment is the need for model personalization and continuous learning. Federated architectures also achieve personalization by enabling local fine-tuning of global models, allowing for adaptation to institutional workflows and evolving imaging protocols without full retraining. This adaptability is essential for maintaining consistent performance over time, especially as clinical environments change due to updated equipment, protocols, or patient demographics.

This study presents a solution to the identified challenges by proposing a fast, accurate, and explainable breast cancer classification system that can be deployed through a web-based application. By utilizing DL techniques, the system enhances diagnostic accuracy while minimizing both false positives and false negatives. It provides real-time AI-driven insights, which help reduce the workload for clinicians and facilitate remote screening in low-resource environments. To build clinical trust, the system incorporates XAI techniques, such as Grad-CAM, which visually highlights the areas of the tumor that contribute to the classification. To protect data privacy, the model utilizes FL, allowing for decentralized training without the need to exchange data. Moreover, the system is designed for personalization and continual learning, ensuring that it can adapt to various clinical contexts and evolving data distributions.

Our proposed methodology follows a structured pipeline that utilizes four independent breast cancer imaging datasets: BreakHis, BUSI, INbreast, and CBIS-DDSM. To address class imbalance and improve learning for the minority class, we implement extensive data augmentation. We use multiple variants of the Swin Transformer to extract spatial features from the images. These features are then combined using an RF meta-learner to increase robustness. To ensure privacy across institutions, we employ federated training. Additionally, we utilize Grad-CAM-based visualizations to provide transparency regarding the model’s decision-making process. The final system is validated across all datasets and will be deployed through a web application called BreastInsight, which offers real-time, interpretable predictions. The key contributions of this research are as follows:

- A proposed privacy-preserving system features enhanced hierarchical model aggregation strategies, allowing secure decentralized training while ensuring data confidentiality across various institutions.
- A comprehensive ensemble learning system is created by combining Swin Transformer variants (Tiny, Small, Base, Large) with an RF meta-learner, improving multi-scale spatial representation and enhancing classification accuracy across various datasets.
- The integration of Grad-CAM provides interpretable visual justifications for each prediction, thereby enhancing transparency and fostering clinician trust in AI-assisted diagnostics.
- The BreastInsight web application is designed for real-time deployment, incorporating tools for explainability, FL updates, and readiness for personalization, which enhances practical clinical integration and long-term usability.

The remainder of this paper is organized as follows: Section 2 reviews related work on DL and federated approaches for diagnosing breast cancer. Section 3 describes the proposed methodology, including preprocessing, feature extraction, and model training. Section 4 presents the results along with an analysis of explainability. Section 5 discusses the key findings, limitations, and future directions of this research. Finally, Section 6 concludes with a summary of the contributions and their clinical implications.

## 2. Related Works

DL has significantly improved breast cancer classification. Most current methods utilize CNNs, enhanced through hybrid architectures, data augmentation, optimization algorithms, and attention-based models. While these approaches often achieve high accuracy, they face practical challenges such as data imbalance, lack of robustness across various domains, dependence on centralized training, and limited explainability for clinical use. This section critically reviews existing methods, highlighting how newer approaches address past limitations and identifying remaining gaps.

### 2.1. Transfer Learning Models

CNN-based systems have been fundamental in the classification of breast cancer. Their primary strength lies in automated feature extraction and the ability to scale to large datasets. For example, Chakravarthy et al. [22] proposed a hybrid feature fusion approach that combines various CNN architectures, including VGG16, ResNet50, and DenseNet121. This method achieved over 98% accuracy on the CBIS-DDSM and INbreast datasets. However, it faced challenges with malignancy detection and required significant computational resources. Wani et al. [23] introduced a hybrid CNN-LGBM system (BC2) that was enhanced by SHAP-based interpretability. This approach reached an accuracy of 98.3% on SEER data, but it required tuning specific to the dataset and struggled with generalization across different institutions. Also, Xiao et al. [24] utilized InceptionV3 for breast cytopathology classification. By implementing image segmentation and fusion for block-level predictions, they achieved an accuracy of approximately 92.01% across four different magnifications on the BreaKHis dataset.

To enhance diagnostic staging, Ajlouni et al. [25] utilized a CNN-SOM system for TNM classification from breast MRI, achieving an impressive staging accuracy of 98%. However, the absence of validation on external datasets limits its clinical relevance. Kollem et al. [26] developed a multi-architecture pipeline using PResNet-34 and VGG16, implementing Twin SVM-RFE for feature reduction. Although they achieved near-perfect accuracy, their approach relied on handcrafted data augmentation and faced challenges regarding scalability. Rajkumar et al. [27] combined CLAHE and HGLCM with DarkNet-53 for histopathology classification, attaining a 95.6% accuracy rate. Yet, this method suffered from inflexible hyperparameter settings and a lack of adaptability. Despite their strengths, CNN-based models often overfit due to dataset-specific distributions and struggle with underrepresented classes. These challenges have led to the implementation of augmentation and optimization techniques.

### 2.2. GAN-Augmented and Optimization-Based Models

To address data imbalance and enhance learning from limited samples, generative and metaheuristic techniques have been introduced. Munteanu et al. [28] utilized GAN-generated synthetic samples within a U-Net and CNN system, achieving an accuracy of 86% on the BUSI dataset. While GANs improved the representation of minority classes, this method was still dependent on the specific dataset and lacked external robustness. Similarly, Jabeen et al. [29] developed a residual CNN using a quantum distribution optimizer and kernel CCA, which reached an accuracy of 96.5% on the INbreast dataset. However, this approach was computationally intensive and required extensive parameter tuning.

Optimization strategies have also concentrated on improving model weights and convergence. Emam et al. [30] incorporated LFR-COA into DenseNet121 using bio-inspired techniques, such as Lévy Flight and opposition-based learning. Their model achieved an impressive accuracy of 99.87% on the DMR dataset; however, it incurred high computational costs and did not support decentralized training. Thakur et al. [31] combined attention-based CNNs with reinforcement learning for semantic segmentation, achieving 99.35% accuracy on the DDSM dataset. Nevertheless, its sensitivity to hyperparameter changes and limited multiclass evaluation affected its reliability. These approaches tackle specific challenges such as imbalance and convergence, but they rarely integrate interpretability, cross-domain adaptation, or privacy guarantees. This gap has led researchers to explore alternatives based on attention mechanisms.

### 2.3. ViT Approaches

ViTs have demonstrated strong potential in breast cancer classification by effectively modeling the global spatial relationships within images. Srivastava et al. [32] compared several variants of ViTs, including MaxViT, CvT, and CrossViT, achieving an impressive accuracy of 92.12% on IDC histopathology data. While ViTs outperformed CNNs in capturing the differences between classes, they often struggled to generalize across different datasets due to their sensitivity to variations in imaging. To tackle the issue of magnification variance, Tariq et al. [33] developed a hybrid approach that combines CNN layers with ViT, resulting in magnification-independent classification and an accuracy of 89.43%. However, the consistency at the subclass level remained weak.

Hybrid ViT models have been developed to integrate both global and local feature extraction. Hayat et al. [34] combined EfficientNetV2 with ViT, achieving an impressive 99.83% accuracy for binary classification and 98.10% for multiclass classification on the BreaKHis dataset. However, they noted issues with overfitting and optimization that were specific to the dataset. Meanwhile, Shiri et al. [35] introduced SupCon-ViT, a supervised contrastive learning model based on ViT, which attained a balanced accuracy of 88.61%. Nonetheless, this model did not undergo evaluation across diverse datasets, raising concerns about its scalability in clinical settings.

Several studies have investigated optimization in the context of DL models. Ayana et al. [36] introduced a ViT model specifically designed for HER2 staging using hematoxylin and eosin (H&E) stained samples, achieving an Area Under the Curve (AUC) of 0.9202. While this approach eliminated the need for expensive immunohistochemical (IHC) staining, it still faced challenges in broader clinical integration. Gutierrez-Cardenas et al. [37] combined ViT features with multi-layer perceptron (MLP) and support vector machine (SVM) classifiers, attaining up to 98% accuracy on the DSMM and INbreast datasets. However, their reliance on external classifiers limited the practical deployment of their model. Baroni et al. [38] optimized the ViT model through normalization and augmentation techniques, but their results varied across different datasets, including BACH, BRACS, and AIDPATH, indicating sensitivity to domain shifts. Similarly, Vanitha et al. [39] developed an External Attention Transformer (EAT) and reported a remarkable 99% accuracy on the BreaKHis dataset, although their validation did not encompass multiple classes. Finally, Sabry et al. [40] employed a multi-resolution ViT with post-processing for whole-slide images, achieving an impressive accuracy of 99.42%. However, this approach required significant computational resources.

### 2.4. Federated DL Approaches

Recent studies have increasingly explored the integration of FL with DL architectures for breast cancer diagnosis. El-Mawla et al. [41] introduced FL-L2CNN-BCDet, a hybrid FL system combining convolutional, attention, and BiLSTM modules, achieving up to 99% accuracy on three mammography datasets (DDSM, INbreast, and Microcalcification). However, their model does not address deployment complexity or regulatory compliance, particularly in low-resource settings. Similarly, Selvakanmani et al. [42] incorporated Grad-CAM into an Inception-V3-based FL model, attaining 99.38% accuracy on BreakHis. Yet, their reliance on a single dataset limits generalizability and undermines the robustness required for real-world clinical deployment.

Furthermore, Suriya et al. [43] combined FL with game-theoretic interpretability using Shapley values on a logistic regression model trained on the WBCD dataset. Despite demonstrating adaptability on tabular data, the lack of imaging inputs restricts clinical relevance in visual diagnostics. In a study, Alsalman et al. [44] employed a federated deep convolutional neural network (DCNN) on BUSI, achieving 98.09% accuracy and highlighting communication efficiency as a core benefit. However, their approach did not incorporate explainability mechanisms or cross-dataset validation. Similarly, Peta et al. [45] implemented a CNN-based FL system on the same dataset, reaching 95.5% accuracy. However, their study suffered from high computational overhead and lacked interpretability, impeding its integration into resource-constrained environments.

In another study, Al-Hejr et al. [46] proposed an explainable FL architecture incorporating CNNs, ensemble models, and a hybrid ViT-CNN design for both binary and multi-class breast cancer classification. Their model achieved 98.65% accuracy (binary) and 97.30% (multi-class) across three clients using patient risk factor data, yet may not scale efficiently in complex multi-institutional setups. In a broader application, Bechar et al. [47] fused FL with transfer learning to detect multiple cancer types. For breast cancer, DenseNet-169 achieved 98.73% accuracy on BreakHis, while FL with DenseNet and E-RNN reached 95.73% on CBIS-DDSM. Their work addressed challenges such as communication overhead, privacy preservation, and data heterogeneity but lacked unified interpretability.

### 2.5. Research Gap and Our Contributions

Earlier CNN-based systems for breast cancer classification faced challenges like limited receptive fields, overfitting, and reliance on manual augmentation, which hindered their scalability in clinical settings. This prompted the development of GAN-based and optimization-driven models that enhanced sample diversity and convergence, but these often lacked interpretability, real-world validation, and compliance with privacy standards. Recently, ViT-based models have emerged as a promising solution for spatial feature extraction and long-range dependency modeling. However, they tend to overfit with limited data, lack privacy-preserving mechanisms, and remain opaque in decision-making. Most studies focusing on ViT and FL emphasize centralized evaluation, neglecting issues like data imbalance, non-IID settings, scalability across institutions, and deployment feasibility.

To address these gaps, we introduced a federated ensemble learning system that combines four Swin Transformer variants using a stacked RF meta-learner. It is trained on decentralized datasets with a privacy-preserving FL strategy and includes personalized fine-tuning. For transparency, we incorporate Grad-CAM-based explanations at both the model and interface levels to provide clinicians with clear visualizations of predictions. Additionally, we’ve developed a real-time web application with security features compliant with GDPR and HIPAA for practical clinical use. Our model has been rigorously validated across five imaging datasets and assessed through ablation studies and paired statistical analyses to highlight the contribution of each component. By integrating Swin Transformer architectures, federated training, XAI, and deployment readiness, our study provides a scalable and trustworthy solution for privacy-compliant breast cancer diagnosis in real-world scenarios.

## 3. Methodology

**Figure 1:**
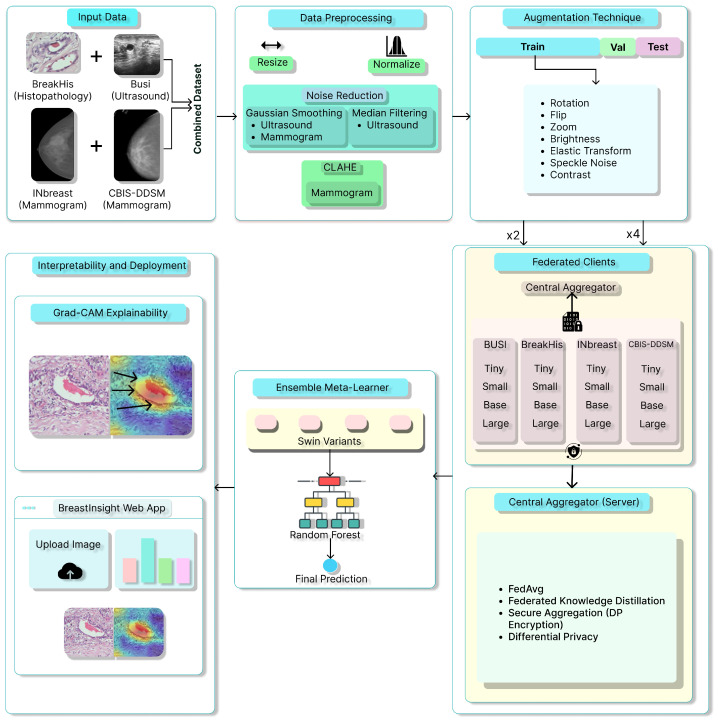
Overview of the proposed BreastSwimFedNetX for robust breast cancer classification. Black arrows in Grad-CAM Explainability subfigure indicate the model’s attention focus.

Figure 1 illustrates the proposed methodology pipeline. This study combines multiple datasets, utilizes thorough preprocessing techniques, and trains various Swin Transformer models. It outlines the training parameters and evaluation metrics. It ensures result interpretability through performance comparisons, Grad-CAM visualizations, and a web application that provides explainable predictions for breast cancer.

### 3.1. Data Description

This study utilizes five publicly accessible datasets: BreakHis [48], BUSI [49], INbreast dataset [50], CBIS-DDSM dataset [51], and a combined dataset [52] created from the integration of these four. These datasets offer complementary imaging modalities for analyzing both morphological and textural features in breast cancer recognition.

**Figure 2:**
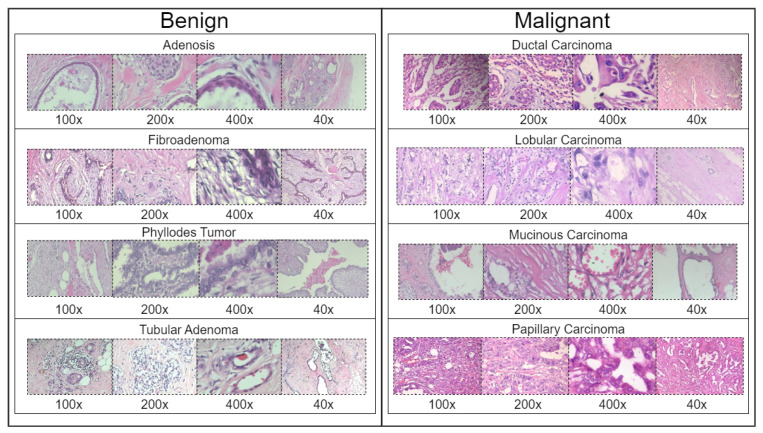
Representative samples from the BreakHis dataset showing benign and malignant breast tissue histopathological images across different magnifications (40×, 100×, 200×, and 400×). The benign samples include Adenosis, Fibroadenoma, Phyllodes Tumor, and Tubular Adenoma, while the malignant samples consist of Ductal Carcinoma, Lobular Carcinoma, Mucinous Carcinoma, and Papillary Carcinoma.

BreakHis is derived from 82 patients and consists of 7909 histopathological images captured using magnifications of 40×, 100×, 200×, and 400×. Figure 2 presents a sample histopathological image representing both benign and malignant categories. The dataset includes 2480 benign samples and 5429 malignant samples. The RGB images have dimensions of 700 × 460 pixels, and the pathologists’ annotations, along with the samples for each image, are obtained through the surgical open biopsy (SOB) method to enhance the representativeness of the samples.

Table 1 summarizes the distribution of classes in the BreakHis dataset across four magnification levels. Ductal Carcinoma is significantly overrepresented, making up the majority of samples at each magnification level, with a peak of 903 images at 100×. In contrast, Adenosis is the least represented subtype, showing a consistent decline in sample size from 114 images at 40× to 106 images at 400×. Other subtypes, such as Fibroadenoma, Papillary Carcinoma, and Phyllodes Tumor, demonstrate relatively balanced distributions. However, Tubular Adenoma and Lobular Carcinoma exhibit moderate decreases in representation as magnification increases. The dataset reaches its maximum size at the 100× magnification, totaling 2081 images, which indicates a potential magnification bias.

**Figure 3:**
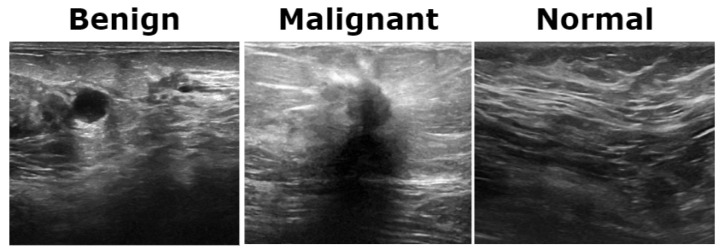
Representative ultrasound images from the BUSI dataset showcasing three classes.

The BUSI dataset consists of 830 breast ultrasound images collected from 600 female patients aged between 25 and 75 years. These images are classified into three diagnostic categories: normal, benign, and malignant (see Figure 3). Additionally, the dataset includes ground truth segmentation masks that aid in accurate lesion delineation. Each image is saved in PNG format with a resolution of 500 × 500 pixels, ensuring consistency for preprocessing and analysis. Unlike histopathological imaging, ultrasound imaging poses challenges such as low signal-to-noise ratios and greater variability.

**Figure 4:**
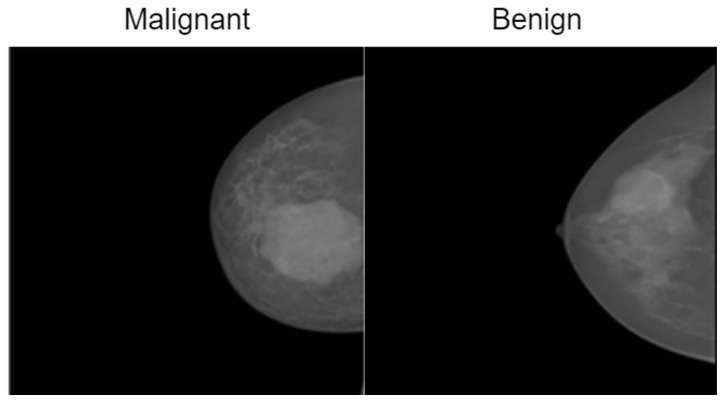
Representative samples from the InBreast dataset showing malignant and normal classes.

The INbreast dataset consists of 7632 full-field digital mammography (FFDM) images collected from 115 patients. Each image has been meticulously annotated by radiology experts to ensure high diagnostic accuracy. The dataset includes standard mammographic views—craniocaudal (CC) and mediolateral oblique (MLO)—for both breasts, although, in cases of mastectomy, only the remaining side is imaged. This dataset captures a variety of clinical scenarios, including screening, diagnostic, and follow-up procedures. For classification purposes, images are labeled as either benign or malignant. Benign cases encompass cysts, fibroadenomas, and calcifications, while malignant cases include tumors and suspicious lesions. What sets INbreast apart is its high-resolution imaging, acquired using a MammoNovation Siemens FFDM system with solid-state amorphous selenium detectors. The images are stored in DICOM format, featuring a resolution of 70 microns per pixel, with dimensions of either 3328 × 4084 or 2560 × 3328 pixels, depending on the compression plate used. Each image is accompanied by detailed XML-based annotations that provide information on lesion contours, types, biopsy results, and BI-RADS categories (3–6). Furthermore, the dataset includes patient metadata such as age, family history, BI-RADS assessments, and breast density classifications according to ACR standards. Figure 4 illustrates representative samples.

**Figure 5:**
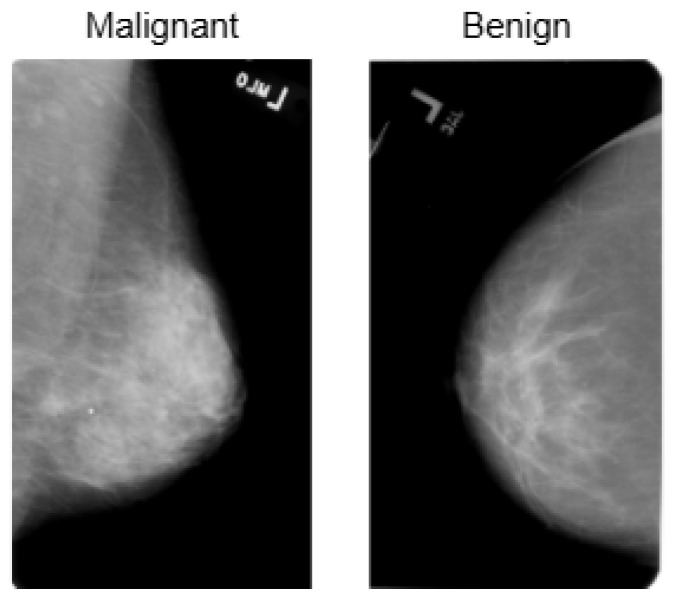
Visualization of the CBIS-DDSM dataset, highlighting its structure and distribution for breast cancer classification.

The CBIS-DDSM consists of 3086 high-resolution mammographic images collected from 892 patients. This dataset is designed to support tasks related to computer-aided detection and diagnosis, specifically focusing on distinguishing between benign and malignant abnormalities. It includes both full-field digital mammograms and cropped regions of interest (ROIs), allowing for precise localization and analysis of suspicious areas. The images are categorized into benign and malignant classes and cover a range of mammographic findings, such as masses, calcifications, and architectural distortions. Each image is annotated with details, including lesion contours, lesion types, and biopsy-confirmed pathology labels. Additionally, BI-RADS assessments and standard mammographic views are provided to ensure clinical consistency. All images are stored in DICOM format, with full-resolution images maintained at a 16-bit depth and cropped ROIs at an 8-bit depth. For preprocessing and compatibility with various models, the images were converted to grayscale PNG format while preserving their original bit depth. ROIs were resized to 598 × 598 pixels to retain pathological detail. Supplementary metadata includes patient demographics, BI-RADS categories, and descriptions of the lesions. Figure 5 presents representative samples from the dataset.

Table 2 shows the class distribution for the BUSI, INbreast, and CBIS-DDSM datasets. A significant class imbalance is evident across all three datasets. In the BUSI dataset, benign cases are predominant, with 487 images, while malignant and normal classes are significantly underrepresented, particularly the normal class, which consists of only 133 images. The INbreast dataset has the largest sample size, where malignant cases account for over two-thirds of the total, with 5112 out of 7632 images, indicating a strong bias toward malignancy. Similarly, the CBIS-DDSM dataset contains more benign (1728) than malignant (1358) samples, although the difference is less pronounced. Importantly, only the BUSI dataset includes a “Normal” category, which highlights its broader clinical scope. The lack of normal samples in the INbreast and CBIS-DDSM datasets may hinder a model’s ability to learn representations of healthy tissue. These discrepancies in class distribution and dataset composition can lead to biased learning and reduced generalization in classification models.

Table 3 illustrates the class distribution of the unified breast cancer dataset, which was created by merging the BreakHis, BUSI, INbreast, and CBIS-DDSM datasets. The analysis reveals a substantial class imbalance, with 12,109 samples (62.8%) classified as malignant and 7165 samples (37.2%) as benign. Notably, BreakHis and INbreast account for the majority of malignant cases, contributing over 85% of all malignant samples. In contrast, BUSI provides a considerably smaller number of samples overall and contributes the least to both classes, particularly for malignant cases, which total only 210 images. This imbalance, which skews toward malignant samples, may lead to biases during the training process. It could particularly affect the model’s ability to recognize benign patterns. Further, the varying distributions across the different datasets reflect differences in data acquisition protocols and labeling standards, complicating model generalization. The underrepresentation of benign cases raises further concerns about the potential for increased false-positive rates.

The integration of these datasets provides several advantages. First, the combined dataset includes a variety of imaging modalities, such as histopathological, ultrasound, and mammographic images. This diversity enables the model to effectively extract both detailed and structural features. Second, by increasing the dataset size, we lower the risk of overfitting, which improves the model’s stability during training. Third, while individual datasets may exhibit class imbalances, merging them leads to a more proportional representation of benign and malignant cases.

### 3.2. Data Preprocessing

Data preprocessing is an essential step in ensuring the uniformity, quality, and reliability of input data for DL models. The preprocessing workflow includes resizing, normalization, contrast enhancement, noise reduction, and data augmentation. These techniques are employed to improve feature extraction and enhance model performance.

To maintain consistency across datasets, all images are resized to a standard dimension of 224×224 pixels while keeping their original aspect ratio. This process standardizes the input dimensions across various imaging modalities. Additionally, normalization is applied to scale pixel intensity values, ensuring numerical stability during training. Min-max normalization rescales pixel values to the range of [0,1], using the Equation (1).

(1) $$I^{\prime }=\frac{I-I_{\min}}{I_{\max}-I_{\min}}$$

where $I^{\prime }$ is the normalized pixel intensity, *I* represents the original intensity, and $I_{\min}$ and $I_{\max}$ denote the minimum and maximum pixel intensities in the image, respectively. Alternatively, z-score normalization is used in cases where mean and variance standardization is required as Equation (2), where μ is the mean pixel intensity, and σ is the standard deviation. This ensures that images have a zero mean and unit variance.

(2) $$I^{\prime }=\frac{I-\mu }{\sigma }$$

Noise reduction techniques are especially important for ultrasound and mammographic datasets, as imaging artifacts can obscure important pathological features. One common method is Gaussian smoothing, which reduces high-frequency noise through a convolution operation Equation (3), where $I^{\prime }\left(x,y\right)$ is the smoothed pixel intensity, and G(i,j) represents the Gaussian kernel as Equation (4), with the degree of smoothing controlled by σ.

(3) $$I^{\prime }\left(x,y\right)=\sum_{i=-k}^{k}\sum_{j=-k}^{k}I\left(x+i,y+j\right)\cdot G\left(i,j\right)$$

(4) $$G\left(i,j\right)=\frac{1}{2\pi \sigma^{2}}e^{-\frac{i^{2}+j^{2}}{2\sigma^{2}}}$$

For ultrasound images, median filtering is applied to suppress speckle noise represented as Equation (5).

(5) $$I^{\prime }\left(x,y\right)=\mathrm{median}\left\{I\left(m,n\right)|\left(m,n\right)\in \mathrm{neighborhood}\;\mathrm{of}\;\left(x,y\right)\right\}$$

For mammographic images, Contrast Limited Adaptive Histogram Equalization (CLAHE) enhances low-contrast regions, improving the visibility of lesions. CLAHE operates based on Equation (6), where *L* represents the number of intensity levels in the image.

(6) $$I^{\prime }=\frac{\left(I-I_{\min}\right)\times \left(L-1\right)}{I_{\max}-I_{\min}}$$

Each dataset undergoes an 80:5:15 stratified split to maintain class balance across the different partitions. Table 4 outlines the dataset-specific augmentation strategies employed in this study. The parameters were selected based on typical acquisition conditions and the visual characteristics of each modality, ensuring that the transformations are clinically reasonable. By introducing controlled variations, this strategy enhances the model’s robustness against both intra-dataset variability and inter-dataset domain shifts. This approach particularly benefits the performance of minority classes while preserving anatomical fidelity.

**Figure 6:**
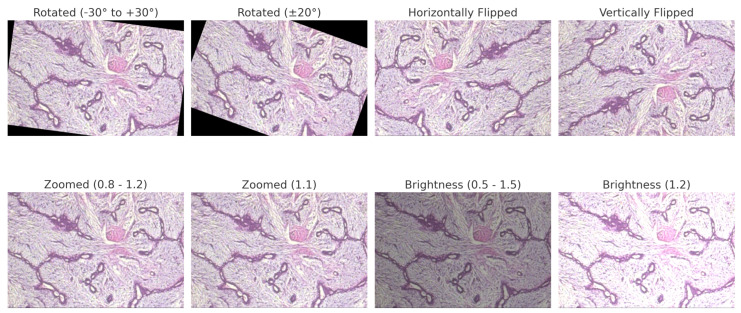
BreakHis preprocessed sample with applied data augmentation techniques, including rotation, flipping, zooming, and brightness adjustment.

**Figure 7:**
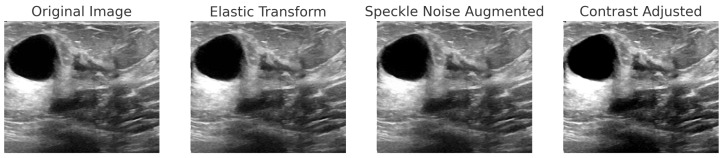
Step-by-step augmentation of a BUSI dataset sample. The original ultrasound image undergoes three transformations: (1) Elastic Transform to introduce slight deformations; (2) Speckle Noise Augmentation to simulate real-world noise variations; (3) Contrast Adjustment to enhance intensity variations.

**Figure 8:**
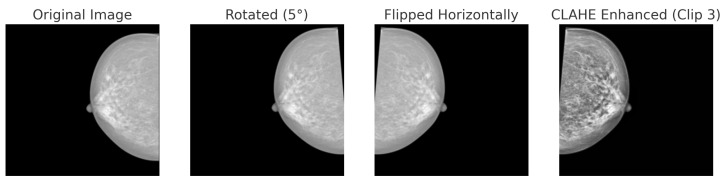
Data augmentation techniques applied to a mammogram image from the INbreast dataset: original, rotated (+5°), horizontally flipped and CLAHE.

**Figure 9:**
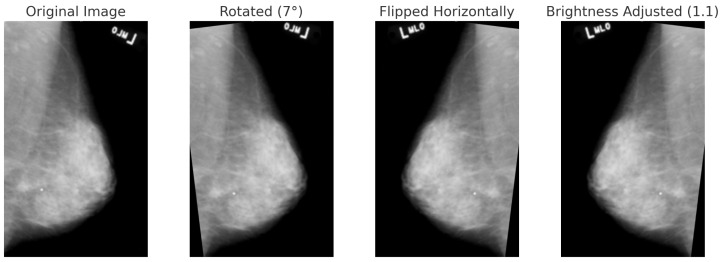
Data augmentation techniques applied to a mammogram image from the CBIS-DDSM dataset: original, rotated (+7°), horizontally flipped, and brightness-adjusted.

For BreakHis, extensive geometric (rotation ± 20°, flips) and photometric (zoom, brightness) transformations were applied to reflect slide orientation variability and staining inconsistencies. BUSI employed elastic deformation, speckle noise, and contrast adjustment to mimic tissue distortion and signal artifacts. INbreast received conservative adjustments to enhance tissue visibility without losing diagnostic fidelity. CBIS-DDSM used mild rotation, flipping, and brightness changes, maintaining lesion structure integrity. The Combined Dataset integrated harmonized augmentations from all sources, ensuring cross-modality consistency while retaining BUSI-specific elastic transformation. The chosen values prioritize generalization while preventing excessive transformations that could alter pathological features or diminish diagnostic relevance. Figure 6, Figure 7, Figure 8 and Figure 9 illustrate sample images from each dataset after the application of these data augmentation techniques.

Table 5 outlines the original class distributions and the corresponding data splits (training, validation, and testing) for all datasets. It also includes two augmentation targets: doubling (×2) and quadrupling (×4) the training set size. This augmentation is particularly beneficial for underrepresented classes, such as Adenosis in BreakHis and Malignant in BUSI, as it enhances feature generalization and reduces the risk of bias during the learning process. The scaling approach is designed to proportionally increase intra-class variability while preserving the integrity of the dataset. The combined dataset consists of 15,419 training samples, which are augmented to 30,838 samples (×2) and 61,676 samples (×4), thereby creating a substantial training base suitable for deep ensemble learning. Such augmentation ensures that the model remains robust against variations across different datasets, magnification levels, and imaging modalities. However, it is important to avoid overfitting to the synthetically expanded samples of rare classes. Therefore, the augmentation strategy has been paired with validation checks to ensure that the distribution remains consistent.

### 3.3. Proposed Model Implementation

#### 3.3.1. Swin Transformers

**Figure 10:**
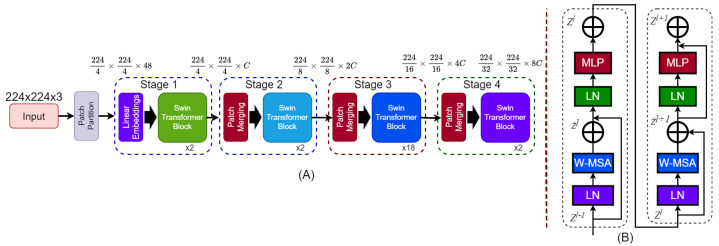
Architecture of the Swin Transformer. (**A**) The hierarchical model structure takes a 224 × 224 × 3 input image, applies patch partitioning and linear embedding, and processes it through four stages with Swin Transformer blocks. Patch merging reduces spatial dimensions and increases feature depth across stages. (**B**) Structure of a Swin Transformer block showing two sub-blocks. Each includes Layer Normalization (LN), Window-based Multi-Head Self-Attention (W-MSA), and a MLP, with residual connections after W-MSA and MLP.

Unlike CNNs, the Swin Transformer is specifically designed for image classification and emphasizes local features. It employs a hierarchical structure with a shifted window self-attention mechanism, effectively balancing computational efficiency and global context [53]. This approach makes it particularly suitable for handling high-resolution images across various datasets. In this study, we examine four variants of the Swin Transformer—Tiny (T), Small (S), Base (B), and Large (L)—to evaluate the trade-offs between accuracy and computational complexity. Swin transformer architecture is depicted in Figure 10.

#### 3.3.2. Technical Details

The Swin Transformer processes input images $I\in R^{H\times W\times C}$ by dividing them into non-overlapping patches of size P×P. Each patch is treated as a “token” and embedded into a high-dimensional feature space using a linear projection, as shown in Equation (7), where $W_{e}$ is the embedding weight matrix, *D* is the embedding dimension, and $Z_{0}$ represents the resulting sequence of patch embeddings. Self-attention in the Swin Transformer is calculated locally within non-overlapping windows, which reduces the computational complexity compared to global self-attention [54]. The self-attention mechanism for a window $X_{\mathrm{window}}$ is defined in Equation (8).

(7) $$Z_{0}=\mathrm{Flatten}\left(I_{\mathrm{patches}}\right)W_{e},\;W_{e}\in R^{(P^{2}\cdot C)\times D}$$

(8) $$\mathrm{Attention}\left(Q,K,V\right)=\mathrm{Softmax}\left(\frac{QK^{⊤}}{\sqrt{d}}+R\right)V$$

Here, Q,K,V are the query, key, and value matrices, respectively. These matrices are computed as shown in Equation (9), where $W_{q},W_{k},W_{v}\in R^{D\times d}$ are learnable weight matrices, and *d* is the dimension of the keys and queries. The term *R* represents the relative position bias, which enhances the model’s ability to encode spatial relationships. By alternating between regular and shifted window partitions, the model ensures connectivity across windows. Each stage in the hierarchical structure operates at progressively lower spatial resolutions and higher feature dimensions. This design mirrors the multiscale feature extraction found in CNNs while retaining the benefits of transformer architectures.

(9) $$Q=X_{\mathrm{window}}W_{q},\;K=X_{\mathrm{window}}W_{k},\;V=X_{\mathrm{window}}W_{v}$$

At each stage of the Swin Transformer, the outputs of the self-attention layers are processed through an MLP to enhance feature representation (10). The MLP consists of two fully connected layers with a GELU nonlinearity applied after the first layer, as described in Equation (10), where $W_{1},W_{2}$ are the weight matrices, $b_{1},b_{2}$ are biases, σ represents the GELU activation function, and *F* is the input feature representation.

(10) $$\mathrm{MLP}\left(F\right)=\sigma \left(FW_{1}+b_{1}\right)W_{2}+b_{2}$$

To train the Swin Transformer for image classification, cross-entropy loss is employed. This loss function ensures that the predicted probabilities align with the ground truth labels. For a dataset with *N* samples and *C* classes, the loss function is defined in Equation (11).

(11) $$L=-\frac{1}{N}\sum_{i=1}^{N}\sum_{j=1}^{C}y_{ij}\log p_{ij}$$

Here, $y_{ij}$ is the ground truth label for the *j*-th class of the *i*-th sample, and $p_{ij}$ is the predicted probability for the same class. This formulation penalizes incorrect predictions proportionally to their confidence.

#### 3.3.3. Stacking Ensemble Architecture

The BreastSwinFedNetX model features a two-level architecture designed to optimize breast cancer classification. At Level 0, it includes four variants of the Swin Transformer: Swin-T, Swin-S, Swin-B, and Swin-L. These variants vary in terms of model depth, embedding dimensions, and the number of attention heads, allowing each model to extract unique features from the input images. This diversity among the base models enhances the ensemble’s ability to capture a broad range of both local and global image features, improving the robustness of BreastSwinFedNetX against variations in the dataset. In the FL context, each Swin Transformer variant acts as a client model trained on disjoint data partitions simulating decentralized hospital environments. This design enables parallel training across local nodes without sharing raw data, thereby preserving patient privacy.

At Level 1, an RF meta-learner aggregates the predictions from the four base models. The RF combines multiple decision trees to reach a final prediction. In this process, the meta-learner learns how to optimally combine the outputs of the base models based on their individual strengths and performance characteristics. It processes the predictions from Swin-T, Swin-S, Swin-B, and Swin-L by appropriately weighting these predictions. The training of BreastSwinFedNetX is staged in two phases. First, the Swin Transformer clients are independently trained in a federated setup using local datasets, where model updates are periodically aggregated using the Federated Averaging (FedAvg) algorithm. This simulates real-world data silos while maintaining privacy compliance. After this initial training phase, the predictions from these models serve as input features for the RF meta-learner, which is trained using cross-validation techniques to prevent overfitting and ensure effective generalization on unseen data. The final output is produced by the meta-learner, which consolidates the predictions from the four base models. This approach utilizes the complementary strengths of the Swin Transformer variants, which effectively capture diverse image features, while the RF meta-learner optimally combines these outputs. This federated ensemble strategy ensures that both local learning and global aggregation are retained in the final decision-making process, balancing model diversity, privacy, and generalization.

**Figure 11:**
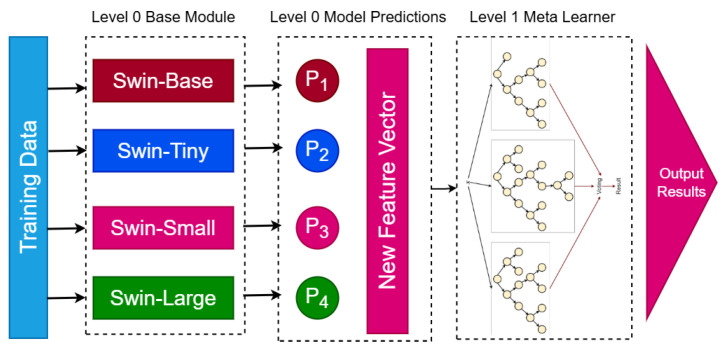
Proposed BreastSwinFedNetX model architecture for multi-class classification. The Level 0 Base Module includes four Swin Transformer variants that generate predictions. These predictions form a New Feature Vector passed to the Level 1 Meta Learner, which produces the final results.

Figure 11 illustrates the architecture of the proposed BreastSwinFedNetX model, showcasing the Swin Transformer variants at Level 0 and the RF meta-learner at Level 1.

The proposed BreastSwinFedNetX classification system is described in Algorithm 1. This system combines variants of the Swin Transformer with an RF stacking ensemble to improve accuracy while maintaining data privacy. The RF classifier effectively manages the heterogeneity and high dimensionality of the combined feature space. By learning decision boundaries through techniques like bagging and feature subspace sampling, it helps reduce variance, prevent overfitting, and ensure robust generalization across different breast cancer subtypes and imaging modalities. Additionally, the interpretability of the RF model and its ability to make non-linear decisions enable it to effectively distinguish between subtle variations in cancer cases.
**Algorithm 1** BreastSwimFedNeXt: Federated Swin Transformer Ensemble for Breast Cancer Classification.**Require:** Distributed datasets $X={\left\{\left(x_{i},y_{i}\right)\right\}}_{i=1}^{N}$ from multiple clients, Swin Transformer variants (Tiny, Small, Base, Large), FL parameters θ   1:Initialize FL parameters θ and set Swin Transformer variants and RF meta-learner   2:**for** each client *i* **do**   3:    Perform local data preprocessing: apply augmentation (rotation, flipping, brightness, Gaussian noise, blur)   4:    Train local Swin Transformer model to extract features:$$f_{\mathrm{swin}}=\mathrm{SwinTransformer}\left(X\right)$$   5:    Apply MHSA:$$\mathrm{Attention}\left(Q,K,V\right)=\mathrm{softmax}\left(\frac{QK^{T}}{\sqrt{d_{k}}}\right)V$$   6:    Optimize with local cross-entropy loss:$$L_{\mathrm{local}}=-\sum y\log\left({\hat{y}}_{\mathrm{local}}\right)$$   7:**end for**   8:Server aggregates model weights using FedAvg:$$W_{\mathrm{global}}=\frac{1}{N}\sum W_{{\mathrm{local}}_{i}}$$   9:Server constructs stacking ensemble: base predictions from all Swin variants → RF meta-learner$$\hat{y}={\mathrm{RF}}^{*}\left(f_{\mathrm{SwinTiny}},f_{\mathrm{SwinSmall}},f_{\mathrm{SwinBase}},f_{\mathrm{SwinLarge}}\right)$$   10:**return** Final prediction $\hat{y}$

### 3.4. Training Settings

All experiments were conducted on a workstation equipped with dual NVIDIA RTX A6000 GPUs, each having 48 GB of VRAM, along with 256 GB of DDR4 RAM and an Intel Xeon Gold 6338 CPU running Ubuntu 22.04 LTS. The training was implemented using PyTorch v1.13.1 with CUDA 11.8, and mixed-precision training was enabled through NVIDIA Apex, which allowed for efficient memory utilization and faster convergence. This setup facilitated the parallel training of high-resolution medical images, providing stability and accelerating the experimentation cycles.

Table 6 summarizes the selected training parameters and their candidate ranges. A batch size of 32 and a learning rate of $1\times 10^{-5}$ were chosen to balance stability and convergence. The AdamW optimizer was selected for its superior generalization, and cosine annealing was used for dynamic learning rate decay. Regularization techniques were applied, including a dropout rate of 0.5, weight decay of $1\times 10^{-4}$, and early stopping with a patience of 5. Warm-up steps (500) and gradient clipping (1.0) were implemented to ensure stable optimization, especially for deeper models. Additionally, mixed precision training and batch normalization enhanced efficiency and convergence. This configuration was validated to support robust model training across various datasets.

### 3.5. Privacy-Preserving FL Setup

In our study, each Swin Transformer variant was assigned to a dedicated client node in the FL system, each trained on a unique, non-overlapping subset of the data to simulate institution-specific learning. This client simulation mimics real-world data silos and allows for model training without centralized data pooling.

**Figure 12:**
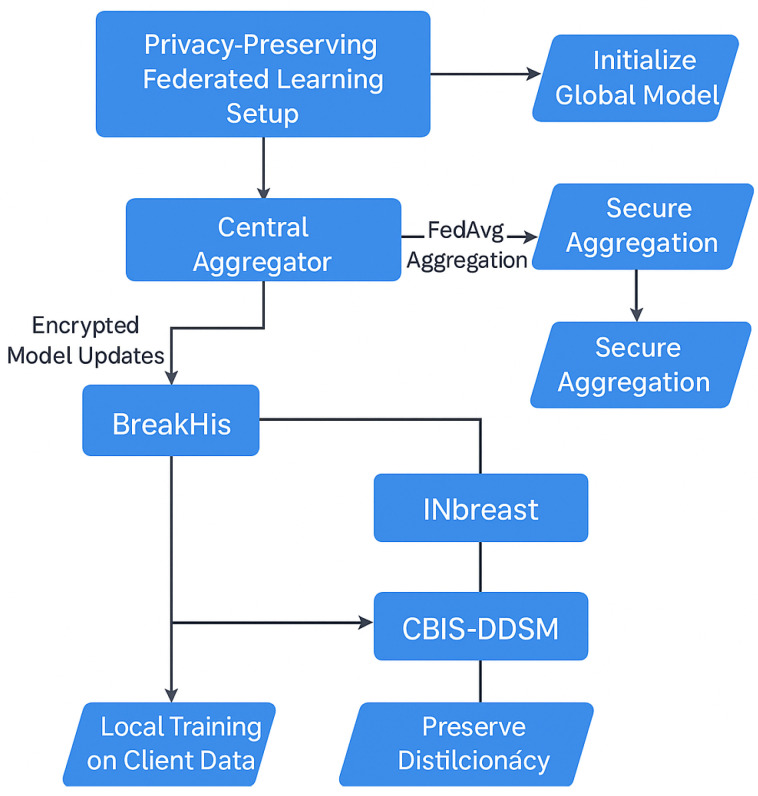
FL setup in BreastSwinFedNetX.

To ensure privacy-compliant model training while utilizing data from multiple institutions, we employed an FL approach for the BreastSwinFedNetX model. The complete FL training workflow is illustrated in Figure 12, which details local training, model encryption, aggregation, and feedback loops that ensure robustness across various clinical domains. Instead of centralizing sensitive medical data, the FL approach allows each dataset to function as a decentralized client node. Local models are trained independently on each client’s data, with only encrypted model updates being shared with a central aggregator. This architecture maintains data sovereignty.

Model training at the client nodes utilized the Swin Transformer variants, which processed imaging data locally and sent encrypted weight updates rather than raw images. These updates were integrated into a global model at the server using the FedAvg algorithm. This configuration ensured that Swin models could collaboratively learn rich feature representations while preserving institutional data privacy.

Each round of FL included three local training epochs, followed by server-side aggregation using the FedAvg algorithm. The training occurred over 50 communication rounds, with every client participating in each round. To ensure balanced contributions from datasets of varying sizes and modalities, we utilized an adaptive weighting mechanism within FedAvg. Clients that generated more stable and informative updates received higher aggregation weights, which helped prevent dominance by data-rich clients, such as BreakHis. To further stabilize training and knowledge sharing across heterogeneous domains, we employed Federated Knowledge Distillation (FKD), in which teacher models trained on richer datasets provided soft-label supervision to clients with limited samples. This soft guidance enabled the Swin variants at weaker nodes to align more effectively with the global distribution. We addressed data heterogeneity by fine-tuning the global model on each local client. This personalization step ensured that the model could adapt to local imaging characteristics while still preserving the shared representations learned from the global federation. To further counter client drift, we also evaluated FedProx regularization but found FKD to be more effective for our study.

Model synchronization can introduce communication overhead, especially during high-frequency FL rounds. To address this issue, we optimized the communication interval by exchanging updates every three local epochs and implemented weight quantization to compress the updates. Furthermore, each Swin client incorporated a local validation loop to ensure early stopping when overfitting was detected, minimizing unnecessary communication rounds. For added security, we integrated secure aggregation using homomorphic encryption (specifically the Paillier scheme) and applied differential privacy with calibrated Gaussian noise (with an ε=1.0) to protect the model parameters. Further, we employed role-based access control (RBAC) and OAuth 2.0 authentication during the web deployment phase to enforce access restrictions.

### 3.6. Evaluation

To evaluate the swine variants reliably, we used 10-fold stratified cross-validation. This method maintains the class proportions across the folds, reducing bias from class imbalance and ensuring that minority classes are consistently represented during both training and validation. Unlike random k-fold cross-validation, stratification prevents the overrepresentation or omission of rare classes in any fold. We chose 10 folds to strike a balance between computational efficiency and evaluation severity, allowing each model to train on 90% of the data and validate on 10%. The final performance metrics were averaged across all folds.

We evaluated the models using four metrics specifically designed for imbalanced medical data: Micro Specificity, Micro F1 Score, MCC, and Precision-Recall AUC (PR AUC). Micro Specificity (Equation (12)) measures the model’s ability to correctly identify negative cases across all classes, which is particularly important in reducing false positives in clinical diagnostics. MCC (Equation (14)) evaluates prediction quality based on the confusion matrix, accounting for true positives (TP), true negatives (TN), false positives (FP), and false negatives (FN), and is robust to class imbalance. Let *C* denote the number of classes, $TP_{i}$ the number of true positives for class *i*, $FP_{i}$ the number of false positives for class *i*, and $FN_{i}$ the number of false negatives for class *i*.

(12) $$\mathrm{Micro}\;\mathrm{Specificity}=\frac{\sum_{i=1}^{C}TN_{i}}{\sum_{i=1}^{C}\left(TN_{i}+FP_{i}\right)}$$

(13) $$\mathrm{Micro}\;F1=\frac{2\times \sum_{i=1}^{C}TP_{i}}{2\times \sum_{i=1}^{C}TP_{i}+\sum_{i=1}^{C}FP_{i}+\sum_{i=1}^{C}FN_{i}}$$

(14) $$\mathrm{MCC}=\frac{TP\times TN-FP\times FN}{\sqrt{\left(TP+FP)(TP+FN)(TN+FP)(TN+FN\right)}}$$

The PR AUC metric quantifies the trade-off between precision and recall across all thresholds and emphasizes the model’s ability to detect positive (malignant) cases, particularly in imbalanced datasets. It is computed from the precision-recall curve and is defined as follows:

$$\mathrm{PR}\;\mathrm{AUC}=\int_{0}^{1}\mathrm{Precision}\left(r\right)\;dr$$

where Precision(r) is the precision at a given recall level *r*, and the integral is typically approximated using the trapezoidal rule over discrete recall points.

## 4. Experimental Results

### 4.1. Proposed Models’ Performance Showcase

Table 7 and Table 8 present a comparative analysis of model performance under two augmentation settings: ×2 and ×4. Across all datasets and models, the ×4 augmentation consistently yields higher scores in every key metric, including specificity, MCC, PR AUC, and F_1_-score. On average, transitioning from ×2 to ×4 results in gains of approximately 0.9 percentage points in specificity, 0.8 in MCC, 0.7 in PR AUC, and 1.1 in F_1_-score. These improvements are particularly significant in MCC and F_1_, as they are more balanced indicators of classification quality in imbalanced medical datasets. For example, in the BreakHis dataset, the BreastSwinFedNetX model experienced an increase in F_1_-score from 98.54 to 99.39 and a rise in MCC from 96.26 to 97.29 when trained with ×4 augmentation. Similarly, the Swin-L model on the Combined dataset showed an MCC improvement from 97.43 to 98.96 and a specificity increase from 96.02 to 97.59. Even in the BUSI dataset, which is known for its noise and low-contrast imaging, the Swin-B model enhanced its specificity from 92.21 to 93.52 and its F_1_-score from 92.11 to 93.25. These consistent improvements underscore the significance of utilizing extensive augmentation when training DL models for breast cancer classification.

Nearly all metrics indicate an improvement with the use of ×4, although a few minor deviations have been noted. For instance, the PR AUC score for BreastSwinFedNetX on the BUSI dataset experienced a slight decline, dropping from 96.67 to 97.93. However, this change is statistically insignificant and can be attributed to natural fluctuations in performance, particularly at high accuracy levels, especially given that the dataset is already close to its maximum performance. Importantly, the absolute scores remain the highest among all competing models. To ensure the reliability of these findings, each experiment was repeated across ten randomized runs using fixed train-validation-test splits. Performance metrics showed consistently low variance, typically within a range of 0.5 to 1.5 percentage points, which demonstrates strong convergence and stability of the model across trials.

**Figure 13:**
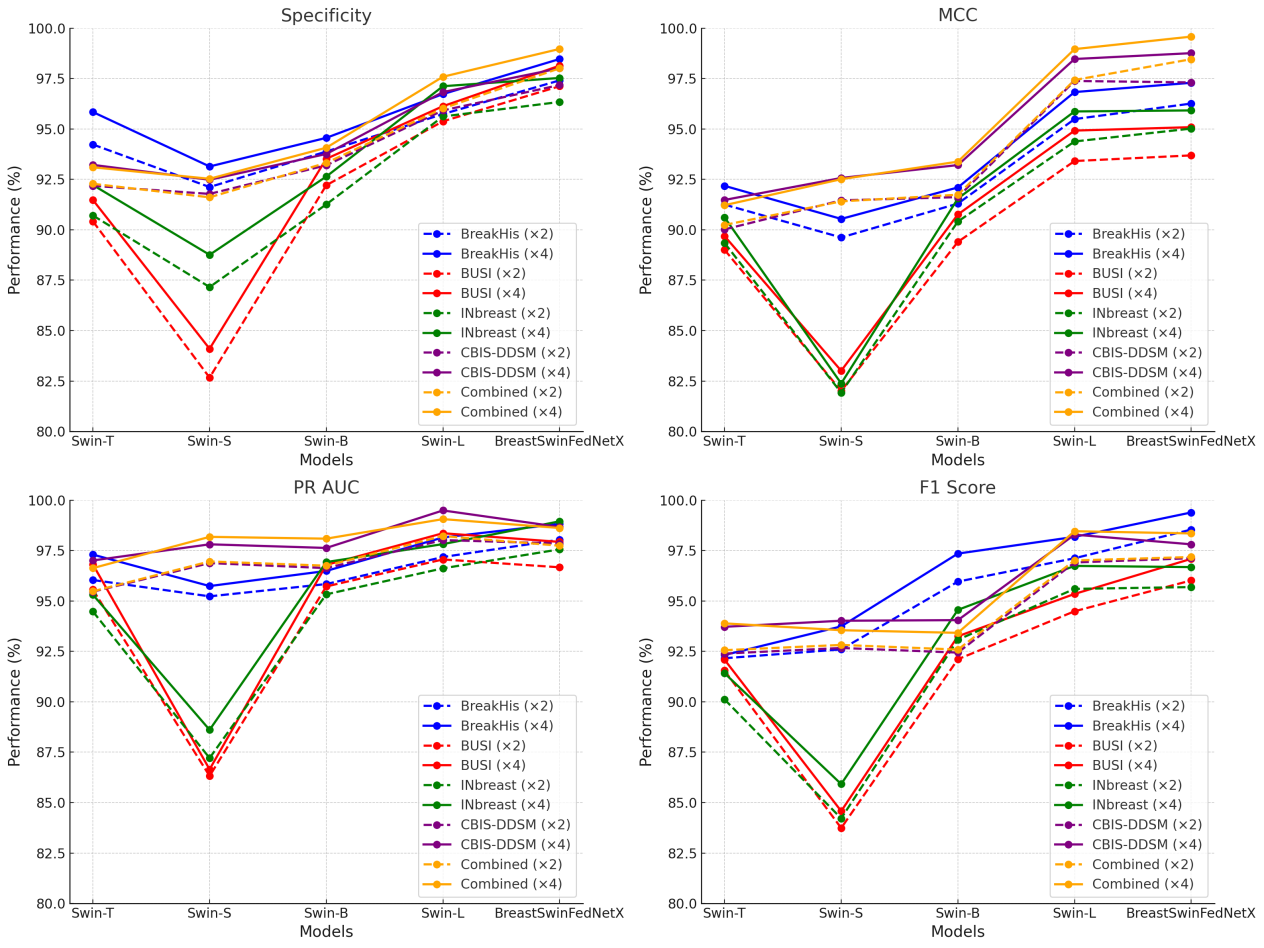
Performance improvement of DL models across datasets before and after data augmentation.

The transition from ×2 to ×4 augmentation leads to improvements across all evaluated metrics and datasets. These benefits are particularly important for detecting minority classes, enhancing robustness to domain shifts, and reducing false positives and negatives in clinical decision-making. Considering the relatively small increase in computational cost alongside the substantial gains in performance and stability, ×4 augmentation emerges as the optimal choice for training scalable and reliable breast cancer diagnostic models. Analyzing the datasets reveals that BreakHis and CBIS-DDSM exhibited high performance even before applying augmentation, showing only moderate improvements afterward. In contrast, the BUSI and Inbreast datasets demonstrated considerable performance gains. The Combined dataset achieved the highest overall improvements, underscoring the effectiveness of utilizing diverse datasets to enhance classification accuracy. Figure 13 illustrates the performance improvements of experimental models trained with ×4 augmentation across different datasets compared to those trained with ×2 augmentation.

### 4.2. Class-Wise Performance Comparison of BreastSwinFedNetX

Table 9 and Table 10 present the class-wise performance analysis of BreastSwinFedNetX across experimental datasets under ×2 and ×4 augmentation, respectively. Across all datasets, the ×4 augmentation strategy consistently enhanced performance for nearly every class in all metrics.

In the BreakHis dataset, improvements in F_1_-scores were seen across every class. For instance, Adenosis—an underrepresented minority class—experienced a substantial increase in its F_1_-score, rising from 96.96 to 99.42. Its specificity also improved, going from 96.91 to 99.50. Similar enhancements were observed in Phyllodes Tumor, where the F_1_-score increased from 95.86 to 98.66, as well as in Ductal Carcinoma, which saw its F_1_-score rise from 96.34 to 99.50. These improvements indicate that the ×4 training setting enhances the model’s ability to generalize across small and morphologically variable classes, which are typically prone to false negatives. All three classes also benefited from augmentation in the BUSI dataset. Notably, the Malignant class improved from a high F_1_-score of 97.55 to 99.22, with the MCC increasing from 96.34 to 99.50. These results suggest that increased sample variability effectively enhanced the model’s understanding of subtle lesion boundaries and irregular echotextures. Consistent improvements were also observed in the CBIS-DDSM and Combined datasets. In the CBIS-DDSM dataset, the F_1_-score for the Benign class rose from 96.18 to 98.99, with the MCC increasing from 95.15 to 98.51. Similarly, in the Combined dataset, both the Benign and Malignant classes achieved an F_1_-score exceeding 98.5 across all four metrics under the ×4 training setting. This confirms that an expanded training distribution improved inter-class discrimination and prediction balance.

Performance improvements were largely consistent across the board, although some subtle shifts were noted. For instance, in the INbreast dataset, the Benign class experienced a slight decrease in the F_1_-score, dropping from 95.94 to 95.51, despite an increase in PR AUC from 98.40 to 99.12. However, these differences were minor and did not affect the overall reliability of the model. The results also indicated greater performance stability across classes. Under ×2 augmentation, the class-wise F_1_-scores in the BreakHis dataset ranged from 94.88 to 97.55, while in the ×4 setting, all classes exceeded 98.1. This reduction in performance variance suggests that ×4 augmentation helps the model learn more consistent feature representations across various imaging modalities. Additionally, the improvements are particularly notable in underrepresented classes, indicating that the augmentation strategy not only boosts overall accuracy but also enhances generalization.

### 4.3. Performance Validation of BreastSwinFedNetX

The BreastSwinFedNetX model, trained on ×4 augmented data, clearly outperforms all baseline and backbone models in both multiclass and binary breast cancer classification tasks. The highest classification scores were achieved on the BreakHis and Combined datasets, respectively, for these two tasks. To validate these impressive results, we conducted a detailed analysis of the training dynamics by examining the learning curves. The analysis supports the effectiveness of BreastSwinFedNetX, particularly when trained on richly augmented data. The BreakHis dataset demonstrates the model’s robustness in handling fine-grained subclass differentiation, while the Combined dataset highlights its efficiency and stability in binary classification scenarios. Furthermore, the learning curves reinforce the notion that the ensemble architecture consistently delivers reliable and clinically relevant performance across various classification settings.

**Figure 14:**
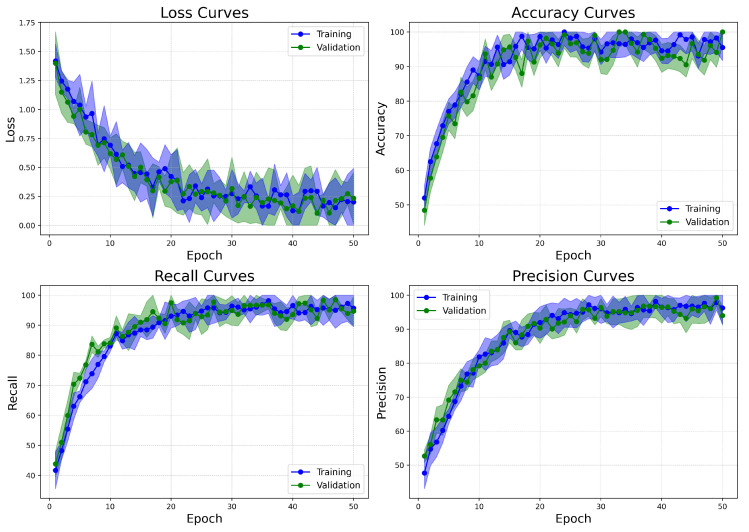
Learning curves of BreastSwinFedNetX model on highest performing multiclass classification dataset.

The learning curves for the BreakHis dataset (Figure 14) demonstrate effective and stable convergence of the model. Both training and validation losses consistently decrease over the 50 epochs, leveling off after epoch 35, with no indications of overfitting. The accuracy rapidly exceeds 98% within the first 15 epochs and remains stable thereafter, highlighting the model’s ability to extract rich and discriminative features from a complex multiclass environment. Recall shows a steady increase, plateauing above 99%, which is particularly important due to the clinical necessity of minimizing false negatives in cancer diagnosis. Precision also exhibits a similar upward trend, exceeding 98% by the final epochs. The small gap between training and validation metrics for loss, accuracy, recall, and precision further supports the model’s generalization capabilities, even across various histopathological subtypes.

**Figure 15:**
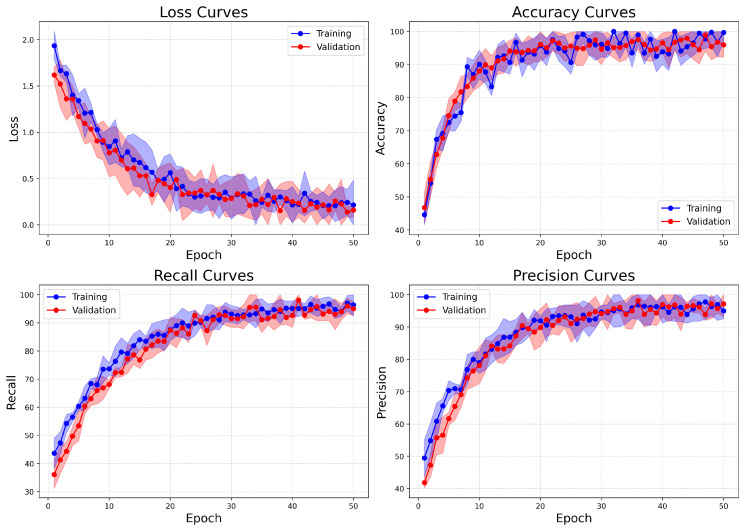
Learning curves of BreastSwinFedNetX model on highest performing binary classification dataset.

On the other hand, the Combined dataset learning curves (Figure 15) show similar patterns of convergence but demonstrate improved stability and faster alignment of metrics. These attributes are likely related to the binary nature of the classification task. The validation loss decreases more steadily and slightly faster compared to BreakHis, indicating clearer class boundaries between benign and malignant categories. Accuracy reaches its peak early and closely aligns between training and validation from epoch 15 onward, signifying reduced overfitting and robust generalization. Both recall and precision metrics rise quickly and stabilize near 98%, with minimal variation between training and validation. This indicates the model’s effective management of false positives and false negatives. In comparison to BreakHis, the Combined dataset exhibits lower variance in metrics across epochs, reflecting the reduced complexity and intra-class variability typically associated with binary classification.

**Figure 16:**
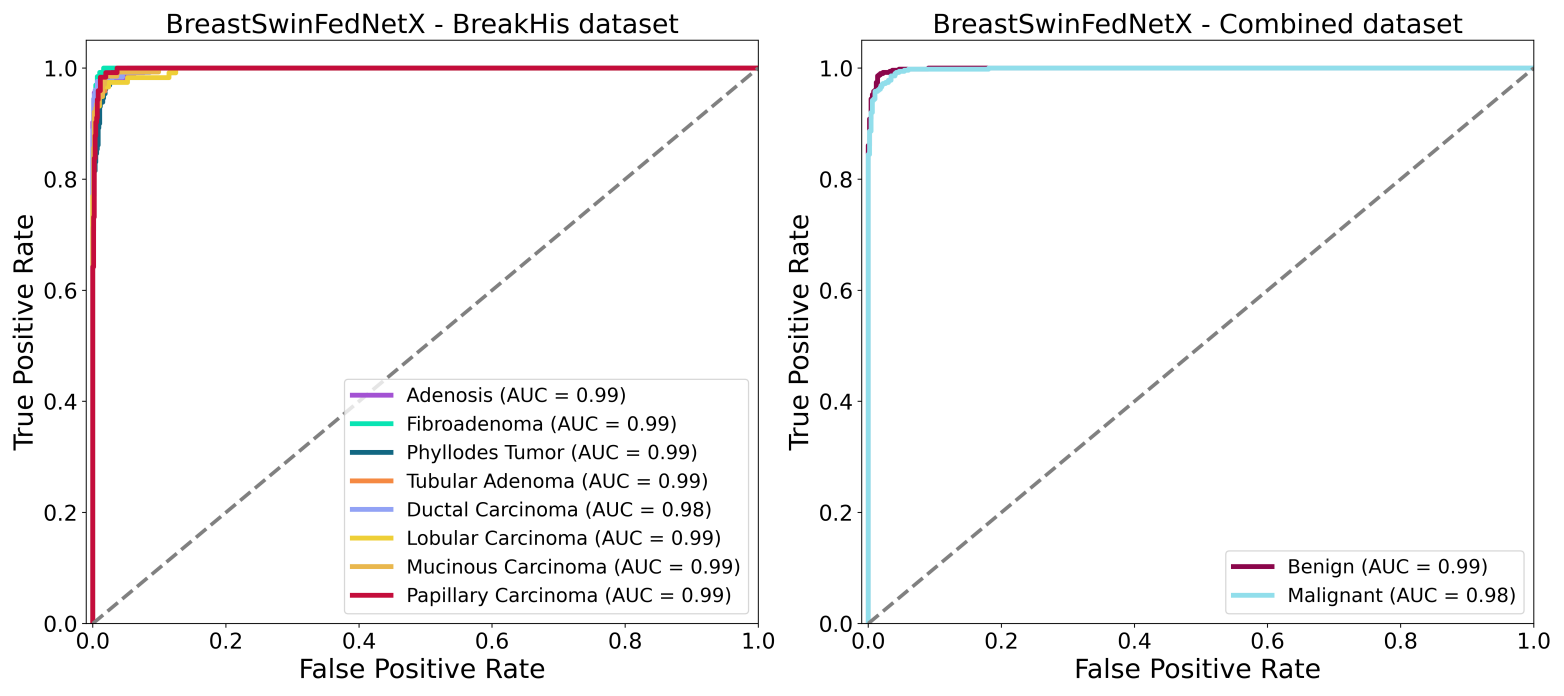
ROC AUC curves of BreastSwinFedNetX model on highest performing multiclass and binary classification dataset.

The ROC-AUC curves shown in Figure 16 further confirm the strong classification performance of the BreastSwinFedNetX model for both multiclass and binary tasks. In the BreakHis dataset (left plot), the model demonstrates exceptional classification abilities for each subtype. All classes— including minority types such as Adenosis and Phyllodes Tumor—achieve an AUC of 0.99, except for Ductal Carcinoma. The nearly perfect AUC values across all classes indicate that the model maintains a high true positive rate while keeping false positives extremely low in the multiclass setting. The tight clustering of the ROC curves in the upper-left quadrant suggests consistent class separation and robustness. In the binary classification (right plot), the model achieves similarly impressive results. The ROC-AUC for the Benign class is 0.99, while the Malignant class records an AUC of 0.98. These scores reflect a highly effective binary decision boundary with minimal overlap between the two classes. Notably, the ROC curves show minimal deviation from the ideal upper-left trajectory. These results validate the generalization strength of BreastSwinFedNetX across both detailed (subtype-level) and broad (benign vs. malignant) classification tasks. The consistently high AUC values across both scenarios indicate that the model is not only accurate but also resilient to performance degradation across different data domains and classification complexities.

**Figure 17:**
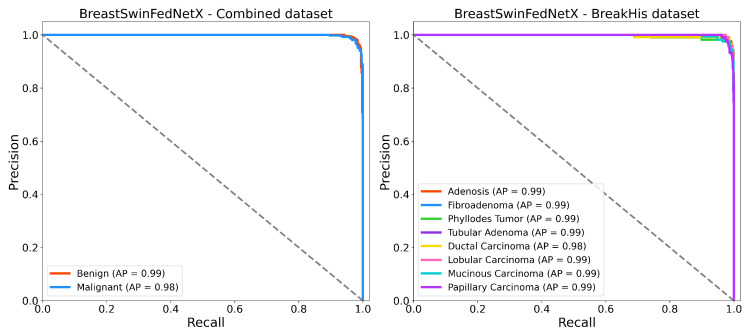
Precision recall curves of BreastSwinFedNetX model on highest performing binary and multiclass classification dataset.

Figure 17 displays the Precision-Recall (PR) curves for BreastSwinFedNetX on the BreakHis and Combined datasets. This provides valuable insights into the model’s discriminative performance, particularly in scenarios with class imbalance. In the multiclass setting, the model consistently achieves high average precision (AP) across all subtypes, with an AP of 0.99 for most classes and 0.98 for Ductal Carcinoma. This indicates that the model effectively handles intra-class variability while maintaining high recall and precision, even for underrepresented subtypes such as Adenosis and Phyllodes Tumor. For the binary classification task using the Combined dataset, the model also delivers strong results, achieving an AP of 0.99 for the Benign class and 0.98 for the Malignant class. The near-horizontal PR curves illustrate high classification confidence and reliable separation between benign and malignant samples. These results highlight the robustness of BreastSwinFedNetX in providing balanced and reliable predictions across both fine-grained and broader classification tasks.

### 4.4. Ablation Study on the Combined Dataset

The ablation study presented in Table 11 shows a consistent improvement in performance across all metrics as the Swin Transformer variants increase in size from Tiny to Large. Notably, the Swin-Large variant outperforms its smaller counterparts, achieving an F1 score and PR AUC of 97.88% and 98.01%, respectively. This indicates that larger and more complex models provide better feature representation. However, the proposed BreastSwinFedNetX outperforms all individual Swin variants by achieving the highest scores in all evaluation metrics. It highlights the benefits of integrating multiple Swin variants rather than using them individually.

Table 12 illustrates the meta-learner’s key role in improving ensemble performance. While traditional learners like Logistic Regression, Decision Tree, and SVM perform well (over 97% in Specificity and F1 Score), RF significantly outperforms them with 99.03% Specificity and 99.11% F1 score, along with a PR AUC of 98.62%. Naive Bayes trails behind, struggling with complex decision boundaries in high-dimensional spaces. These findings demonstrate RF’s ability to capture intricate feature interactions and enhance prediction stability, making it the most effective meta-learner in the architecture.

Table 13 demonstrates the improvements in BreastSwinFedNetX’s performance achieved through successive preprocessing techniques. Initially, with only resizing, the model achieved 93.24% specificity and 95.16% F1 score. The addition of normalization resulted in enhancements of 0.87% in the F1 score and 1.24% in PR AUC. Further improvements were observed with the introduction of Gaussian noise, particularly in the MCC, which increased from 91.79% to 93.14%. Contrast stretching further elevated the performance metrics, with the F1 score reaching 97.22%. Ultimately, the complete preprocessing pipeline produced the best results. It is an improvement of up to 8.23% in MCC and 3.19% in PR AUC compared to minimal preprocessing. These findings confirm that a well-designed preprocessing pipeline can greatly enhance model accuracy and robustness.

### 4.5. Model Explainability

**Figure 18:**
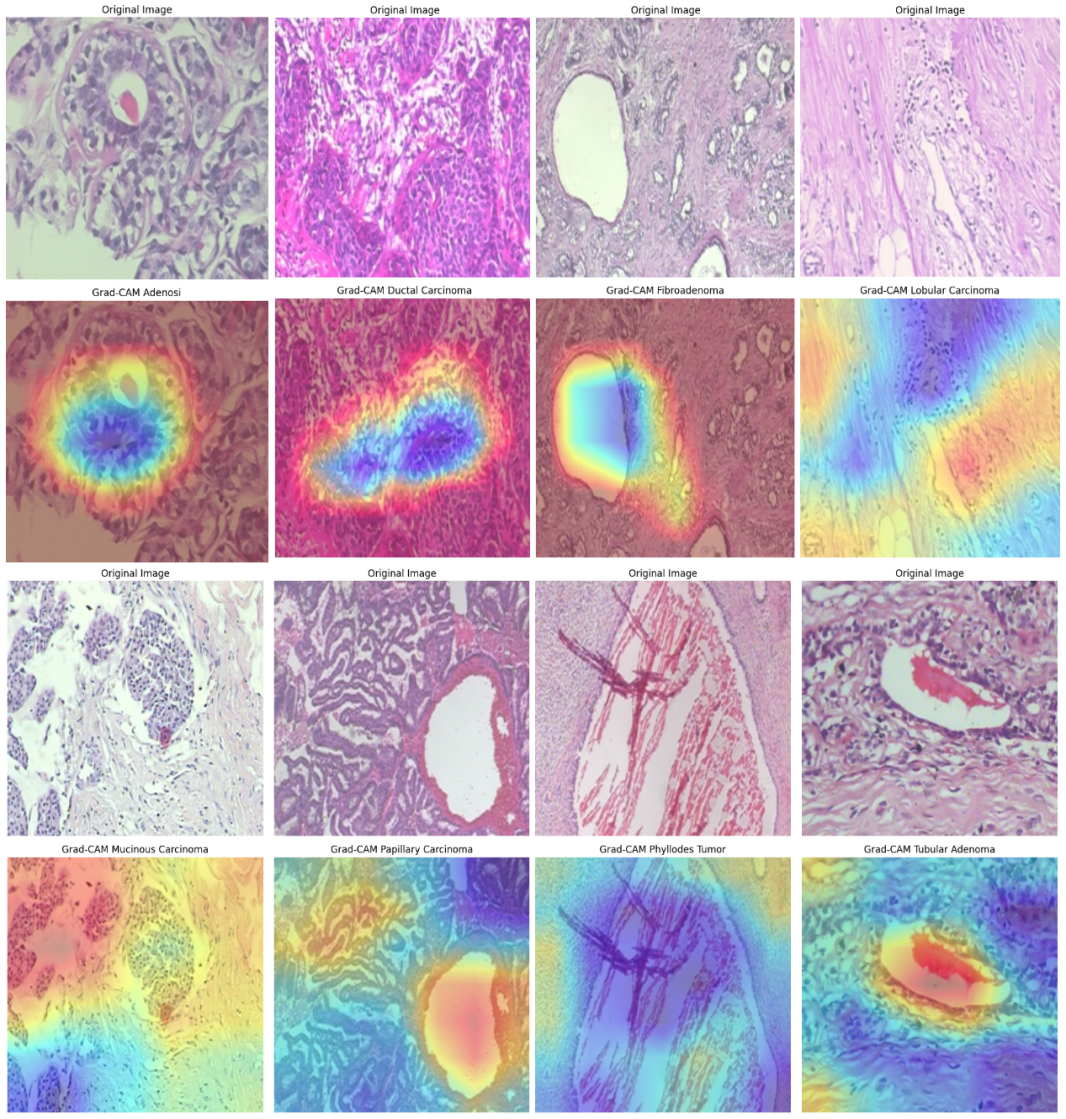
Grad-CAM visualizations on the BreakHis dataset, illustrating model attention in breast cancer classification. Warmer colors (red/yellow) indicate regions of high activation, highlighting critical features used in decision-making.

We further integrated Grad-CAM into the BreastSwinFedNetX pipeline to provide visual explanations for the model’s decisions across all datasets. Grad-CAM highlights the important regions in input images that influence predictions, enhancing model trust and facilitating clinical validation. The Grad-CAM visualizations on the BreakHis dataset (see Figure 18) illustrate precise localization of the model’s attention on morphologically distinct tumor regions. For malignant subtypes, such as ductal and mucinous carcinoma, activations concentrated on dense cellular structures and irregular tissue clusters—areas that pathologists typically associate with cancerous growths. In benign classes like fibroadenoma and tubular adenoma, the model’s focus shifted to localized, well-circumscribed glandular formations, reflecting their less aggressive nature. Notably, minority classes, including adenosis and phyllodes tumor, still demonstrated clear and targeted attention, showcasing the model’s strong generalization despite class imbalance. However, some heatmaps displayed dispersed activation, particularly in overlapping benign subclasses, indicating minor classification uncertainty and the potential for further refinement.

**Figure 19:**
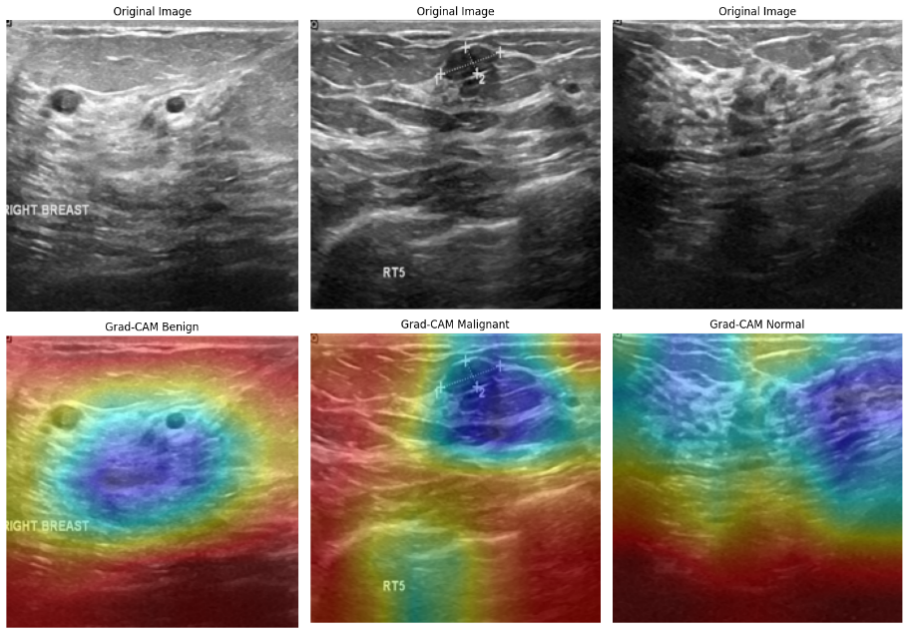
Grad-CAM visualizations of BUSI dataset images, highlighting model focus areas for benign, malignant, and normal cases.

Figure 19 illustrates the Grad-CAM outputs for the BUSI dataset, showcasing the model’s behavior in classifying ultrasound images. In cases of malignancy, the model displayed strong activation around irregular hypoechoic regions, which aligns with typical sonographic patterns associated with malignant tumors. In contrast, benign tumors attracted attention to softer, more homogeneous areas, while normal cases exhibited diffuse, low-intensity heatmaps, indicating the absence of focal masses. The model’s areas of focus closely resemble the zones observed clinically, which enhances confidence in AI-driven decision support. However, the occasional mild overlap between the attention regions for benign and normal cases may contribute to borderline misclassifications—a known challenge in ultrasound-based CAD systems. Despite this, the model demonstrates effective spatial discrimination, particularly for malignant lesions.

**Figure 20:**
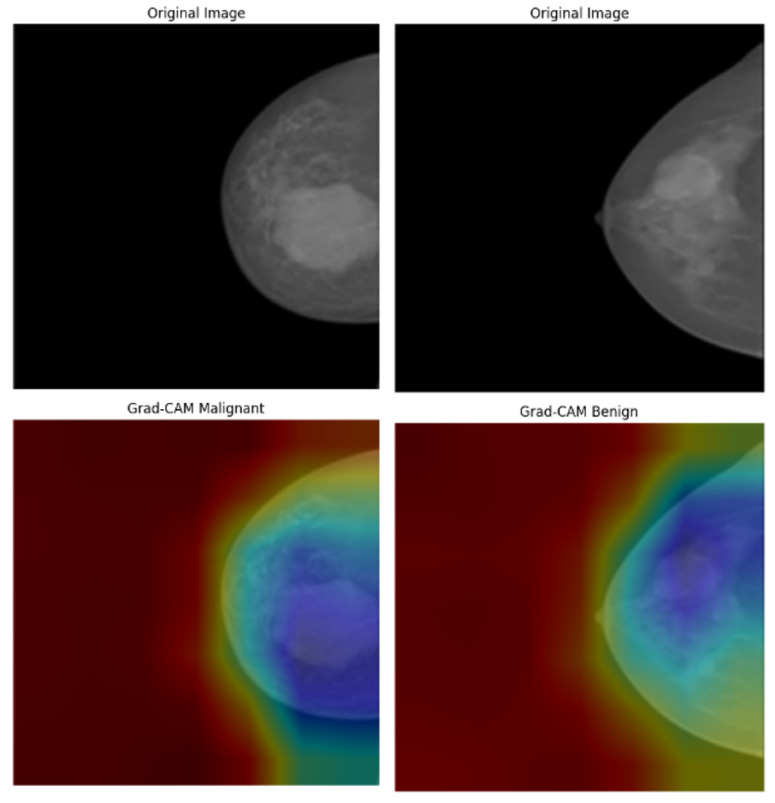
Grad-CAM visualizations of INbreast mammograms highlighting model attention for malignant and benign cases.

In the analysis of the INbreast dataset (Figure 20), the Grad-CAM results provide valuable insights into how the model responds to subtle variations in mammographic density. In malignant cases, the heatmaps are centered around spiculated masses and asymmetric densities, which are key indicators in radiological diagnosis. Conversely, benign cases exhibit lower-intensity activation that is slightly dispersed, reflecting the less suspicious characteristics of benign calcifications or fibroadenomas. The model successfully avoids generating false-positive attention in the background tissue, indicating a well-calibrated feature extractor. Importantly, the distinction between the attention patterns for benign and malignant cases enhances the model’s diagnostic clarity, with visual outputs aligning well with radiologists’ expectations in mammography interpretation.

**Figure 21:**
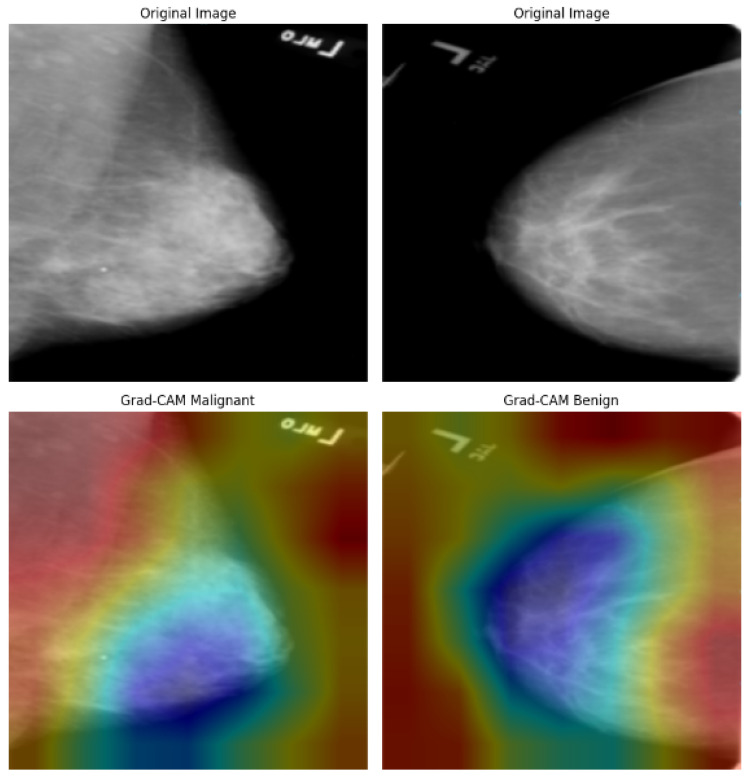
Grad-CAM visualizations of malignant and benign cases from the CBIS-DDSM dataset.

Figure 21 shows the Grad-CAM predictions for the CBIS-DDSM dataset. In images classified as malignant, there is strong activation in areas with dense, irregular tumor margins and spiculated cores, highlighting the model’s effectiveness in identifying cancerous features. In contrast, benign cases reveal broader, less intense activation in well-defined, non-spiculated structures—characteristics typically associated with non-malignant findings. The differences in heatmap intensity and distribution between benign and malignant categories confirm the model’s interpretive accuracy. However, some benign cases exhibit minor peripheral activations, suggesting that the model may be sensitive to nearby tissue textures, which could occasionally result in false positives. Overall, the heatmaps demonstrate clinically consistent patterns.

Across all datasets, Grad-CAM demonstrates that BreastSwinFedNetX focuses on regions that align with clinical hallmarks: spiculated masses observed in mammography, dense nuclei identified in histopathology, and hypoechoic nodules seen in ultrasound. The model’s ability to accurately localize pathology-specific areas, even in the presence of class imbalance and variability across modalities, highlights its interpretative stability. However, instances of dispersed or peripheral activations in benign or low-contrast images indicate that there is room for improvement, particularly through modality-specific fine-tuning.

Table 14 presents a quantitative assessment of the interpretability of Grad-CAM visualizations using three key metrics: Activation Area, Edge Density, and CAM Noise Ratio. Lower values for these indicators indicate more focused, less noisy, and interpretable heatmaps. Among all the models evaluated, BreastSwinFedNetX consistently demonstrates the best interpretability with the lowest scores. This suggests that its Grad-CAM outputs are more spatially compact and less cluttered, effectively highlighting important cancer regions without activating irrelevant background features. In contrast, the individual Swin variants exhibit significantly higher values in all three metrics. These higher values indicate broader and noisier heatmaps that may obscure clinically relevant insights. A clear trend emerges across the Swin variants, showing a decline in interpretability as model complexity decreases. This reinforces the idea that deeper Swin variants provide more focused attention than their smaller counterparts. However, even Swin-Large is substantially outperformed by the federated ensemble model.

### 4.6. FL Evaluation Across Experimental Datasets During Training

The comparative evaluation of FL-based training and centralized training, as shown in Table 15, indicates consistent improvements in classification performance across all datasets. The most significant F1 score gains were observed in the BUSI dataset (+1.09%) and the INbreast dataset (+1.12%). These datasets contained fewer samples and benefited from cross-institutional knowledge transfer. Moderate improvements were also recorded for the CBIS-DDSM dataset (+0.65%) and the Combined Dataset (+0.94%). Even the well-balanced BreakHis dataset experienced a slight improvement of +0.20%.

Although FL provided measurable performance improvements, it also led to increased training time due to communication overhead among the federated nodes (see Table 16). For example, the Combined Dataset took 7.3 h to train, while centralized training only required 6.4 h. This additional time is primarily due to the need for synchronization among multiple participating institutions.

During federated training, we monitored synchronization delays and straggler effects across all participating nodes. Notably, the INbreast and BUSI datasets exhibited occasional straggler behavior due to increased local training times and greater variability in imaging formats and resolutions. However, these delays were addressed by implementing adaptive synchronization intervals and asynchronous update buffering. This approach allowed the server to proceed with partial updates while waiting for slower clients. As a result, the overall training process remained stable without significant degradation in convergence or performance. In the case of the Combined dataset setup, which was larger, we observed minimal synchronization lag due to effective load balancing and periodic checkpointing. These findings suggest that the proposed BreastSwinFedNetX is resilient to common challenges of synchronization in real-world multi-institutional settings.

### 4.7. BreastInsight Application

To bridge the gap between AI-driven breast cancer diagnostics and real-world clinical applications, we developed BreastInsight, a web-based application that operates on a privacy-preserving FL infrastructure. This platform allows clinicians to securely upload breast histopathology, ultrasound, or mammogram images and receive real-time, interpretable predictions generated by the BreastSwinFedNetX model. By utilizing FL, BreastInsight facilitates collaborative model training across multiple institutions without the need to share raw patient data, addressing crucial concerns related to privacy and ensuring compliance with healthcare regulations such as GDPR and HIPAA.

BreastInsight operates on a scalable client-server architecture. Its front end, developed with React.js, offers a user-friendly interface for uploading images, viewing prediction probabilities, and interpreting visual explanations based on Grad-CAM. The backend utilizes FastAPI and Flask microservices to manage image preprocessing, secure inference, and communication with cloud-based GPU infrastructure, such as AWS or Azure. For institutions that require strict data governance, the system supports on-premise deployment, allowing for local inference without the need for an internet connection. The FL strategy facilitates distributed model updates, ensuring that BreastSwinFedNetX continuously evolves across diverse imaging datasets while preserving user privacy. Security is integrated throughout the system architecture. During training, only encrypted model updates—rather than raw data—are shared, utilizing homomorphic encryption and secure aggregation protocols. RBAC, in conjunction with OAuth 2.0, ensures that only authorized personnel can access sensitive data. Additionally, differential privacy techniques introduce statistical noise to further protect model updates from reverse engineering or data leakage, significantly reducing the risk of breaches.

**Figure 22:**
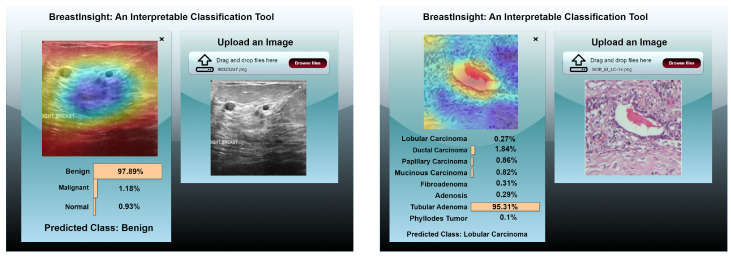
BreastInsight user interface during inference. The clinicians upload an image and the global BreastSwinFedNetX model takes in the subsequently processed image. Class probabilities for each instance are displayed in the form of horizontal bars.

BreastInsight is designed for real-time clinical use, processing each image in under two seconds. It first applies a series of preprocessing steps, including resizing, normalization, denoising, and segmentation, before sending the image to the model. The system is compatible with widely used datasets, such as BreakHis (histopathology), BUSI (ultrasound), and INbreast/CBIS-DDSM (mammography). It outputs confidence scores along with predictions to assist in clinical decision-making. To enhance interpretability, BreastInsight employs Grad-CAM visualizations (see Figure 22), which highlight the regions of the image that influenced the model’s predictions. Clinicians can also compare the AI-generated outputs with patient history, positioning BreastInsight as a decision-support tool rather than a standalone diagnostic solution.

**Figure 23:**
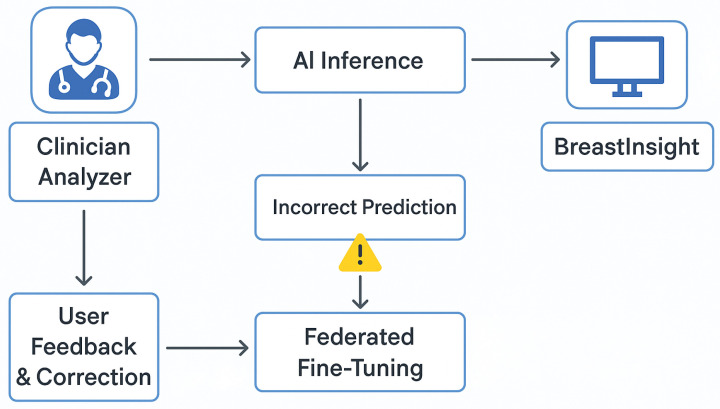
Flowchart of BreastInsight’s feedback and error correction mechanism.

The system is designed for high-throughput scenarios, allowing multiple concurrent inference requests without noticeable latency. Its adaptable infrastructure enables seamless deployment in both cloud and on-premise environments, making it ideal for large-scale hospital networks and clinical research collaborations. To ensure continuous model improvement and clinical reliability, BreastInsight incorporates a user feedback and error correction mechanism. This allows medical professionals to provide real-time input. When a clinician encounters an incorrect or uncertain prediction, the application enables them to flag the case and optionally add notes or diagnostic insights. These flagged samples are securely logged in a manner that preserves privacy and can be stored locally or on institutional servers without transmitting raw image data. During scheduled federated retraining cycles, these flagged cases are utilized to fine-tune the model by incorporating human feedback into the learning process. This helps the model adapt to evolving clinical nuances and become more robust against edge cases and rare subtypes. Additionally, BreastInsight implements a confidence-aware thresholding system that prioritizes low-confidence predictions for review, which minimizes the risk of diagnostic errors. As illustrated in Figure 23, the feedback pipeline features a structured interface for clinician input, secure logging, and privacy-compliant updates to the global BreastSwinFedNetX model through the FL system.

### 4.8. Comparison of Proposed and Existing Models

While many studies have shown impressive results in breast cancer classification, they often reveal critical gaps in dataset diversity, interpretability, and privacy compliance. Our proposed model, BreastSwinFedNetX, addresses these issues directly. Table 17 compares existing DL methods for breast cancer classification. Models like those by Hayat et al. [34] and Emam et al. [30] achieved high accuracies, but their evaluations were limited to specific datasets, raising concerns about their generalization. Many models rely on complex optimizations without adequately assessing robustness across various datasets. Although transformer-based models like SupCon-ViT [35], ViT [37], and MaxViT [32] can capture long-range dependencies, their performance is often inconsistent due to dataset-specific biases and a lack of evaluation in non-IID settings. Additionally, interpretability is frequently overlooked in these approaches. In contrast, BreastSwinFedNetX not only achieves high accuracy across all benchmark datasets but also shows consistent generalization across diverse imaging modalities. Its performance remains stable even on smaller datasets like BUSI and INbreast, due to its FL-based training pipeline, which effectively manages heterogeneous and distributed data.

Unlike previous models that focus solely on accuracy or interpretability, our model incorporates XAI through Grad-CAM, providing clinicians with heatmaps to verify and interpret predictions. This feature is often missing in state-of-the-art approaches. While some competing models face issues like training instability and high computational costs, BreastSwinFedNetX offers a privacy-aware design. Using FL eliminates the need for centralized data aggregation, keeping sensitive patient data local to each medical institution, making it suitable for privacy-regulated environments. Although our approach introduces some computational complexity due to transformer-based ensembles and FL synchronization overhead, these are intentional choices aimed at ensuring robustness, generalization, and interpretability. The trade-offs are justified by the model’s real-time performance and high accuracy, making it ready for deployment in clinical settings.

## 5. Discussion

BreastSwinFedNetX represents a scalable, interpretable, and privacy-preserving system designed for breast cancer classification. As clinical AI continues to evolve, it is essential for such systems to balance performance with transparency, adaptability, and trustworthiness to create a sustainable impact in real-world applications. The impressive performance of BreastSwinFedNetX is attributed to its combination of hierarchical Swin Transformers and ensemble learning strategies. The use of shifted window self-attention in the Swin Transformer variants allows for effective local texture recognition and global context modeling, which are crucial for identifying multiscale morphological patterns in histopathology, ultrasound, and mammography images. By stacking different Swin variants and aggregating their outputs through an RF meta-learner, the model achieves strong generalization across various imaging modalities. This ensemble approach minimizes overfitting and enhances decision reliability, particularly in multiclass classification tasks such as BreakHis. Moreover, FL adds an extra layer of adaptability by enabling decentralized training on non-IID datasets. Each participating institution can contribute to the model’s learning without revealing raw data, ensuring privacy and compliance with HIPAA and GDPR regulations. The implementation of adaptive FedAvg weighting and local fine-tuning further strengthens generalization across diverse data domains.

Our augmentation pipeline improves resilience against class imbalance, scanner variation, and imaging noise. The techniques used in this pipeline simulate diverse acquisition conditions while preserving the anatomical fidelity of lesions. Methods such as MixUp and CutMix were crucial in promoting variability among minority class learning. These augmentations significantly enhanced model convergence and inter-dataset robustness, especially with heterogeneous datasets like BUSI and CBIS-DDSM. To ensure stability, we selected augmentation parameters within clinically plausible limits. This design choice prevented the model from overfitting to unrealistic transformations or artifacts, thereby supporting strong performance in real-world conditions. Moreover, BreastSwinFedNetX addresses the black-box nature of DL by utilizing Grad-CAM, which visualizes the spatial attention on input images. These interpretable heatmaps allow clinicians to visually verify the model’s predictions, thereby fostering greater confidence in its accuracy. Additionally, uncertainty quantification through Monte Carlo Dropout identifies ambiguous cases, prompting manual review and reducing the risk of misdiagnosis.

To assess the effectiveness of BreastSwinFedNetX, we conducted a paired *t*-test (α=0.005) using the BreakHis, BUSI, INbreast, and CBIS-DDSM datasets. The results in Table 18 and Table 19 show significant improvement for BreastSwinFedNetX, particularly against weaker Swin variants like Swin-T and Swin-S, especially in higher-resolution datasets like BreakHis and INbreast. However, its advantage decreases when compared to Swin-B and Swin-L in lower-resolution and less diverse datasets like BUSI.

In the BreakHis dataset, BreastSwinFedNetX outperformed most Swin variants with significant *p*-values (below 0.005) for metrics like Specificity, F1 Score, and PR AUC. For instance, comparisons against Swin-T showed low *p*-values, indicating a strong advantage. Although *p*-values against Swin-B were higher (0.693 for Specificity), they suggest that Swin-B performed similarly to the ensemble, with not all metrics showing significant improvements. On the BUSI dataset, significant gains were noted over Swin-T (p<0.005 for all metrics) and some improvements over Swin-S, particularly in PR AUC (0.072) and MCC (0.021), indicating a trend of superiority. However, comparisons with Swin-L and Swin-B did not yield significant differences (p>0.05), implying competitive performance in this smaller ultrasound-based dataset, likely due to its distinct texture and feature distribution. In the INbreast dataset, BreastSwinFedNetX again demonstrated clear superiority over Swin-T and Swin-S, with *p*-values between 0.002 and 0.009, showing effective generalization across high-resolution mammographic data. Differences against Swin-B and Swin-L were less pronounced (e.g., p=0.612 for Specificity vs. Swin-B). The CBIS-DDSM results echoed these trends, with BreastSwinFedNetX consistently outperforming Swin-T and Swin-S (p<0.005 for most metrics), and significant improvements noted in MCC (0.004 vs. Swin-T). While Swin-L showed no significant gap (p>0.1), Swin-B had marginal significance in MCC (p=0.006), indicating that BreastSwinFedNetX provides a meaningful performance boost in diverse datasets like CBIS-DDSM.

Looking ahead, integrating concept-based explanations can enhance multi-layer interpretability and improve clinician engagement. However, BreastSwinFedNetX faces deployment challenges due to the high parameter count of the Swin-L model, which significantly increases GPU usage and inference latency. To support deployment in hospital settings, techniques such as quantization, model pruning, and tensor decomposition should be explored. Moreover, the BreastInsight platform is designed for scalability, accommodating both cloud-based and on-premise deployments. Integration with Electronic Health Record (EHR) systems through FHIR-based APIs is planned to ensure compliance with healthcare IT standards. Furthermore, the system aims to achieve real-time inference with a latency of under two seconds, along with role-based access, to maintain security and practicality in clinical use. The model’s high-throughput inference pipeline is optimized for batch processing and is scalable to large hospital networks.

BreastSwinFedNetX demonstrates strong performance on benchmark datasets; however, challenges arise in real-world clinical settings due to domain drift and out-of-distribution (OOD) scenarios. Future research should explore Continuous Federated Learning (CFL) to enable the model to adapt incrementally to new imaging devices, protocols, and patient populations. Furthermore, the robustness of models to noise and artifacts has not been thoroughly investigated. It is crucial to evaluate performance under conditions such as adversarial perturbations, low-quality scans, or mislabeled data to ensure safe deployment. Implementing certified defenses or using robustness-aware loss functions may enhance stability. Additionally, model fairness and clinical equity are vital in the deployment of AI systems. While the datasets utilized include various imaging modalities, they often lack demographic annotations. Future research should explore whether model performance differs based on factors such as age, ethnicity, or equipment manufacturers to prevent algorithmic bias. Moreover, the importance of human-AI collaboration cannot be overstated. While BreastSwinFedNetX can provide valuable support, it should not replace clinical judgment. Future usability studies involving radiologists and pathologists are necessary to evaluate how visual explanations and confidence metrics affect decision-making in practice.

## 6. Conclusions

This study presents a federated ensemble model that combines variations of the Swin Transformer with an RF meta-learner to facilitate privacy-preserving breast cancer classification. The model achieved state-of-the-art performance across various datasets, including histopathology, ultrasound, and mammography, while adhering to GDPR and HIPAA regulations. The federated approach allows for collaborative learning without the need for centralized data sharing, thereby enhancing the model’s ability to generalize across non-IID medical data. The BreastInsight web application demonstrates real-time inference, XAI-based interpretability, and uncertainty-aware prediction, all of which enhance clinical trust and usability. However, the model’s computational complexity may limit its applicability in low-resource environments. Future work will focus on enabling edge deployment through model compression and on-device FL inference. Additionally, improving aggregation methods with Graph Neural Networks and securing training with differential homomorphic encryption will further enhance scalability and security. Finally, validating the model across multiple institutions will be crucial to ensuring its clinical adoption and real-world impact. Ultimately, BreastSwinFedNetX paves the way for developing trustworthy, privacy-aware AI systems for breast cancer diagnostics.

## Figures and Tables

**Table 1:** Class distribution of the BreakHis dataset across different magnification factors.

Subtype	40×	100×	200×	400×
Adenosis	114	113	111	106
Fibroadenoma	253	260	264	237
Phyllodes Tumor	109	121	108	115
Tubular Adenoma	149	150	140	130
Ductal Carcinoma	864	903	896	788
Lobular Carcinoma	156	170	163	137
Mucinous Carcinoma	205	222	196	169
Papillary Carcinoma	145	142	135	138
Grand Total	1995	2081	2013	1820

**Table 2:** Class distribution across the BUSI, INbreast, and CBIS-DDSM datasets.

Class	BUSI	INbreast	CBIS-DDSM
Normal	133	–	–
Benign	487	2520	1728
Malignant	210	5112	1358
Total	830	7632	3086

**Table 3:** Class distribution in the combined dataset.

Dataset	Benign	Malignant
BreakHis	2480	5429
BUSI	437	210
INbreast	2520	5112
CBIS-DDSM	1728	1358
Total	7165	12,109

**Table 4:** Augmentation parameters for each dataset.

Dataset	Technique	Range of Values	Selected
BreakHis	Rotation	$-30^{\circ }$ to $+30^{\circ }$	$\pm 20^{\circ }$
	Horizontal Flip	Yes/No	Yes
	Vertical Flip	Yes/No	Yes
	Zoom	0.8–1.2	1.1
	Brightness Adjustment	0.5–1.5	1.2
BUSI	Elastic Transform	Alpha = 30–50	40
	Speckle Noise	0.01–0.05	0.02
	Contrast Adjustment	0.8–1.5	1.2
INbreast	Rotation	$-10^{\circ }$ to $+10^{\circ }$	$\pm 5^{\circ }$
	Horizontal Flip	Yes/No	Yes
	CLAHE	Clip Limit 2–4	3
CBIS-DDSM	Rotation	$-15^{\circ }$ to $+15^{\circ }$	$\pm 7^{\circ }$
	Horizontal Flip	Yes/No	Yes
	Brightness Adjustment	0.8–1.2	1.1
Combined	Rotation	$-15^{\circ }$ to $+15^{\circ }$	$\pm 10^{\circ }$
	Horizontal Flip	Yes/No	Yes
	Contrast Adjustment	0.8–1.5	1.2
	Brightness Adjustment	0.8–1.5	1.1
	Elastic Transform	Alpha = 30–50	40

**Table 5:** Class distribution and data splits across all datasets.

Dataset	Class	Original Count	Training (80%)	Target Training (×2)	Target Training (×4)	Validation (5%)	Testing (15%)
BreakHis	Adenosis	444	355	710	1420	22	67
	Fibroadenoma	1014	811	1622	3244	51	152
	Phyllodes Tumor	453	362	724	1448	23	68
	Tubular Adenoma	569	455	910	1820	28	86
	Ductal Carcinoma	3451	2760	5520	11040	173	518
	Lobular Carcinoma	626	500	1000	2000	31	95
	Mucinous Carcinoma	792	633	1266	2532	40	119
	Papillary Carcinoma	560	448	896	1792	28	84
	Total	8909	7324	14648	29296	445	1340
BUSI	Normal	133	106	212	424	7	20
	Benign	437	349	698	1396	22	66
	Malignant	210	168	336	672	11	31
	Total	780	623	1246	2492	40	117
INbreast	Benign	2520	2016	4032	8064	126	378
	Malignant	5112	4089	8178	16356	256	767
	Total	7632	6105	12210	24420	382	1145
CBIS-DDSM	Benign	1728	1382	2764	5528	86	260
	Malignant	1358	1086	2172	4344	68	204
	Total	3086	2468	4936	9872	154	464
Combined Dataset	Benign	7165	5732	11464	22928	358	1075
	Malignant	12109	9687	19374	38748	606	1816
	Total	19274	15419	30838	61676	964	2891

**Table 6:** Extended training parameters for model training with candidate ranges and selected values.

Parameter	Candidates / Range	Selected Value
Batch size	{16, 32, 64}	32
Initial learning rate	{$1\times 10^{-6}$, $1\times 10^{-5}$, $1\times 10^{-4}$}	$1\times 10^{-5}$
Optimizer	{SGD, Adam, AdamW}	AdamW
Learning rate scheduler	{Step decay, Exponential decay, Cosine annealing, Cyclic}	Cosine annealing
Epochs	{15, 20, 35, 50}	50
Weight decay	{$1\times 10^{-6}$, $1\times 10^{-5}$, $1\times 10^{-4}$, $1\times 10^{-3}$}	$1\times 10^{-4}$
Warm-up steps	{0, 100, 500, 1000}	500
Dropout rate	{0.0, 0.25, 0.5}	0.5
Gradient clipping	{None, 0.5, 1.0, 2.0}	1.0
Mixed precision training	{Enabled, Disabled}	Enabled
Early stopping patience	{3, 5, 10}	5
Batch normalization	{Yes, No}	Yes

**Table 7:** Performance metrics comparison across different models on augmentation (×2).

Dataset	Metric	Swin-T	Swin-S	Swin-B	Swin-L	BreastSwinFedNetX
BreakHis	Specificity (%)	94.23 ± 1.4	92.12 ± 2.0	93.88 ± 1.6	95.74 ± 1.0	97.41 ± 1.1
	MCC (%)	91.25 ± 1.3	89.62 ± 2.1	91.30 ± 1.9	95.49 ± 1.1	96.26 ± 1.0
	PR AUC (%)	96.04 ± 1.6	95.23 ± 1.7	95.84 ± 1.8	97.18 ± 0.9	98.04 ± 0.8
	F1 Score (%)	92.16 ± 1.7	92.60 ± 1.3	95.96 ± 2.0	97.12 ± 0.8	98.54 ± 1.1
BUSI	Specificity (%)	90.42 ± 1.5	82.67 ± 2.5	92.21 ± 2.2	95.38 ± 1.6	97.12 ± 0.9
	MCC (%)	89.01 ± 1.6	82.01 ± 2.3	89.40 ± 2.6	93.41 ± 1.0	93.69 ± 0.9
	PR AUC (%)	95.56 ± 1.3	86.32 ± 1.9	95.72 ± 1.7	97.06 ± 0.8	96.67 ± 0.7
	F1 Score (%)	91.55 ± 1.5	83.74 ± 2.0	92.11 ± 1.9	94.49 ± 1.3	96.02 ± 1.1
INbreast	Specificity (%)	90.71 ± 1.6	87.16 ± 2.5	91.26 ± 2.3	95.62 ± 1.1	96.34 ± 1.0
	MCC (%)	89.35 ± 1.7	81.92 ± 1.8	90.41 ± 2.1	94.38 ± 1.1	95.02 ± 0.7
	PR AUC (%)	94.48 ± 1.6	87.21 ± 1.6	95.33 ± 1.6	96.62 ± 1.0	97.56 ± 0.9
	F1 Score (%)	90.12 ± 1.8	84.22 ± 2.0	93.08 ± 2.4	95.60 ± 1.3	95.69 ± 0.8
CBIS-DDSM	Specificity (%)	92.18 ± 1.3	91.78 ± 1.7	93.20 ± 2.1	95.92 ± 1.2	97.18 ± 0.9
	MCC (%)	90.02 ± 1.5	91.46 ± 1.8	91.62 ± 2.3	97.38 ± 1.2	97.31 ± 0.6
	PR AUC (%)	95.46 ± 1.2	96.88 ± 1.8	96.63 ± 2.0	98.02 ± 0.9	97.85 ± 0.8
	F1 Score (%)	92.39 ± 1.6	92.67 ± 2.0	92.44 ± 2.5	96.91 ± 1.1	97.12 ± 0.9
Combined	Specificity (%)	92.28 ± 1.2	91.61 ± 2.0	93.32 ± 2.0	96.02 ± 1.3	98.01 ± 1.0
	MCC (%)	90.24 ± 1.6	91.41 ± 1.9	91.74 ± 2.2	97.43 ± 1.0	98.46 ± 0.6
	PR AUC (%)	95.48 ± 1.3	96.96 ± 1.7	96.75 ± 1.9	98.22 ± 0.8	97.74 ± 0.8
	F1 Score (%)	92.56 ± 1.5	92.82 ± 2.2	92.59 ± 2.5	97.01 ± 1.2	97.18 ± 1.0

**Table 8:** Performance metrics comparison across different models on augmentation (×4).

Dataset	Metric	Swin-T	Swin-S	Swin-B	Swin-L	BreastSwinFedNetX
BreakHis	Specificity (%)	95.84 ± 1.12	93.14 ± 1.35	94.56 ± 1.23	96.73 ± 0.94	98.47 ± 0.51
	MCC (%)	92.18 ± 1.48	90.54 ± 1.72	92.10 ± 1.66	96.83 ± 1.15	97.29 ± 0.67
	PR AUC (%)	97.30 ± 1.33	95.74 ± 1.41	96.49 ± 1.25	98.15 ± 1.08	98.82 ± 0.48
	F1 Score (%)	92.32 ± 1.51	93.74 ± 1.29	97.35 ± 1.38	98.18 ± 1.17	99.39 ± 0.45
BUSI	Specificity (%)	91.48 ± 1.63	84.10 ± 1.74	93.52 ± 1.49	96.13 ± 1.36	98.14 ± 0.61
	MCC (%)	89.69 ± 1.54	83.01 ± 1.95	90.76 ± 1.84	94.92 ± 1.23	95.09 ± 0.76
	PR AUC (%)	96.81 ± 1.47	86.65 ± 1.59	96.79 ± 1.31	98.36 ± 1.12	97.93 ± 0.53
	F1 Score (%)	92.09 ± 1.35	84.58 ± 1.51	93.25 ± 1.44	95.35 ± 1.32	97.09 ± 0.47
INbreast	Specificity (%)	92.22 ± 1.89	88.76 ± 1.88	92.64 ± 1.92	97.12 ± 1.07	97.53 ± 0.89
	MCC (%)	90.62 ± 1.72	82.38 ± 1.91	91.55 ± 1.79	95.87 ± 1.06	95.92 ± 0.72
	PR AUC (%)	95.31 ± 1.53	88.61 ± 1.44	96.93 ± 1.60	97.82 ± 1.14	98.95 ± 0.59
	F1 Score (%)	91.42 ± 1.61	85.92 ± 1.68	94.56 ± 1.49	96.74 ± 1.34	96.68 ± 0.62
CBIS-DDSM	Specificity (%)	93.22 ± 1.27	92.48 ± 1.45	93.76 ± 1.38	96.83 ± 1.23	98.01 ± 0.41
	MCC (%)	91.48 ± 1.51	92.57 ± 1.43	93.21 ± 1.55	98.47 ± 1.01	98.76 ± 0.11
	PR AUC (%)	97.01 ± 1.12	97.81 ± 1.08	97.63 ± 1.19	99.49 ± 0.97	98.67 ± 0.14
	F1 Score (%)	93.72 ± 1.43	94.02 ± 1.36	94.05 ± 1.48	98.29 ± 1.06	97.81 ± 0.39
Combined	Specificity (%)	93.10 ± 1.41	92.53 ± 1.28	94.07 ± 1.37	97.59 ± 1.18	98.97 ± 0.36
	MCC (%)	91.22 ± 1.60	92.51 ± 1.49	93.38 ± 1.41	98.96 ± 1.08	99.58 ± 0.31
	PR AUC (%)	96.63 ± 1.38	98.18 ± 1.27	98.09 ± 1.34	99.06 ± 0.93	98.62 ± 0.35
	F1 Score (%)	93.89 ± 1.32	93.55 ± 1.22	93.42 ± 1.51	98.46 ± 1.13	98.35 ± 0.29

**Table 9:** Classification report of BreastSwinFedNetX for all datasets on ×2 augmented training data.

Dataset	Class	Specificity (%)	MCC (%)	PR AUC (%)	F1 Score (%)
BreakHis	Adenosis	96.91	97.03	96.56	96.96
	Fibroadenoma	95.68	96.51	96.72	95.10
	Phyllodes Tumor	96.60	95.02	95.85	95.86
	Tubular Adenoma	95.58	95.00	96.32	95.28
	Ductal Carcinoma	95.65	97.58	96.55	96.34
	Lobular Carcinoma	96.11	94.92	95.30	94.88
	Mucinous Carcinoma	95.96	96.61	96.50	96.89
	Papillary Carcinoma	96.23	96.93	95.52	97.30
BUSI	Normal	95.84	95.26	95.31	96.58
	Benign	96.57	96.38	95.48	95.44
	Malignant	94.91	96.34	97.75	97.55
INbreast	Benign	97.49	94.87	98.40	95.94
	Malignant	96.81	95.73	97.08	94.23
CBIS-DDSM	Benign	96.91	95.15	94.67	96.18
	Malignant	95.21	97.40	95.97	96.38
Combined	Benign	95.71	96.53	97.34	97.03
	Malignant	96.19	97.18	97.36	96.82

**Table 10:** Classification report of BreastSwinFedNetX for all datasets on ×4 augmented training data.

Dataset	Class	Specificity (%)	MCC (%)	PR AUC (%)	F1 Score (%)
BreakHis	Adenosis	99.50	99.19	98.44	99.42
	Fibroadenoma	98.34	99.16	99.48	98.22
	Phyllodes Tumor	99.52	98.29	99.50	98.66
	Tubular Adenoma	98.51	98.78	99.50	98.15
	Ductal Carcinoma	98.44	99.20	99.38	99.50
	Lobular Carcinoma	98.71	98.38	98.01	98.69
	Mucinous Carcinoma	99.35	98.23	99.49	99.06
	Papillary Carcinoma	99.50	98.95	98.47	99.37
BUSI	Normal	98.22	98.70	98.34	98.63
	Benign	99.24	98.82	98.76	98.36
	Malignant	98.52	99.50	99.37	99.22
INbreast	Benign	98.11	96.50	99.12	95.51
	Malignant	96.79	94.08	98.34	97.98
CBIS-DDSM	Benign	99.59	98.51	98.28	98.99
	Malignant	98.31	99.46	99.25	99.39
Combined	Benign	98.95	98.46	99.50	99.38
	Malignant	99.47	99.61	99.49	98.61

**Table 11:** Performance comparison of individual Swin variants versus BreastSwinFedNetX.

Model Variant	Specificity (%)	F1 Score (%)	PR AUC (%)	MCC (%)
Swin-Base	95.48	95.61	95.79	94.22
Swin-Small	96.73	96.85	96.91	95.78
Swin-Tiny	97.28	97.35	97.49	96.52
Swin-Large	97.93	97.88	98.01	97.17
BreastSwinFedNetX	98.97	98.35	98.62	98.35

**Table 12:** Impact of meta-learner choice on final performance.

Meta-Learner	Specificity (%)	F1 Score (%)	PR AUC (%)	MCC (%)
Logistic Regression	97.12	97.20	97.38	96.42
Decision Tree	97.67	97.74	97.81	96.96
K-Nearest Neighbors	96.84	96.91	97.05	95.70
Naive Bayes	95.52	95.68	95.81	94.12
Support Vector Machine	97.23	97.31	97.44	96.38
RF	98.97	98.35	98.62	98.35

**Table 13:** Effect of preprocessing techniques on BreastSwinFedNetX performance.

Preprocessing Configuration	Specificity (%)	F1 Score (%)	PR AUC (%)	MCC (%)
Resizing Only	93.24	95.16	94.02	90.87
+ Normalization	94.01	96.03	95.26	91.79
+ Noise Injection (Gaussian, σ = 0.01)	94.67	96.71	96.13	93.14
+ Contrast Stretching	95.28	97.22	97.03	94.05
All Combined	98.97	98.35	98.62	98.35

**Table 14:** Quantitative evaluation of Grad-CAM outputs based on heatmap compactness and noise metrics on the combined dataset.

Model	Activation Area (%)	Edge Density (%)	CAM Noise Ratio
BreastSwinFedNetX	11.6	19.8	0.085
Swin-Large	19.2	27.1	0.158
Swin-Small	22.0	27.6	0.181
Swin-Base	24.1	29.3	0.217
Swin-Tiny	25.4	31.0	0.211

**Table 15:** F1 Score comparison between FL-based and centralized models.

Dataset	FL-Based (%)	Centralized (%)	Difference (%)
BreakHis	99.39	99.19	+0.20
BUSI	97.09	96.86	+1.09
INbreast	96.68	95.56	+1.12
CBIS-DDSM	97.81	97.16	+0.65
Combined Dataset	99.02	98.08	+0.94

**Table 16:** Training time and communication overhead comparison.

Dataset	Training Time (FL)	Training Time (Centralized)	Communication Overhead
BreakHis	5.2 h	4.8 h	Low
BUSI	6.1 h	5.2 h	Moderate
INbreast	5.8 h	5.0 h	Moderate
CBIS-DDSM	5.0 h	4.6 h	Low
Combined Dataset	7.3 h	6.4 h	High

**Table 17:** Existing work and proposed method result comparison for breast cancer classification.

Ref.	Method	Dataset	Result (%)	Limitations
[29]	CNN	INbreast	96.50	Overfitting, dataset limitations
[37]	ViT	INbreast	98.52	External classifier limits deployment
[41]	FL-L2CNN-BCDet	INbreast	98.33	No deployment or compliance strategy
[29]	ResCNN	INbreast	96.50	High computational cost; complex tuning
[24]	Inception-V3	BreakHis	92.00	Class imbalance, lacks explainability
[39]	EAT	BreakHis	99.00	High complexity, data dependency
[34]	EfficientNetV2 + ViT	BreakHis	98.10	Overfitting risk
[32]	MaxViT	BreakHis	92.12	Dataset diversity, scalability
[42]	FL + InceptionV3	BreakHis	99.38	Single dataset; poor generalization
[28]	GAN + CNN	BUSI	86.00	Dataset dependence; lacks robustness
[33]	CNN + ViT	BUSI	89.43	Weak subclass performance
[35]	SupCon-ViT	BUSI	88.61	No diverse dataset validation
[44]	FL + DCNN	BUSI	98.09	No XAI; lacks cross-dataset validation
[45]	CNN-based FL	BUSI	95.50	No interpretability; high resource needs
[30]	LFR-COA-DenseNet121-BC	CBIS-DDSM	98.97	Optimization sensitivity, dataset limitations
[22]	FHDF	CBIS-DDSM	98.83	Dataset diversity and interpretability
[47]	FL + DenseNet	CBIS-DDSM	95.73	No unified XAI; communication cost
Proposed—BreastSwinFedNetX				
		BreakHis	99.34 ± 0.42	
		BUSI	98.03 ± 0.55	
Ours	BreastSwinFedNetX	INbreast	98.89 ± 0.65	Computational complexity
		CBIS-DDSM	98.58 ± 0.06	

**Table 18:** Statistical significance comparison of BreastSwinFedNetX model on BreakHis and BUSI using paired *t*-test (α=0.005).

Comparison	BreakHis (*p*-Values)				BUSI (*p*-Values)			
	Specificity	F1 Score	PR AUC	MCC	Specificity	F1 Score	PR AUC	MCC
vs. Swin-L	0.002	0.008	0.007	0.041	0.612	0.121	0.158	0.244
vs. Swin-T	0.001	0.004	0.053	0.076	0.003	0.003	0.005	0.003
vs. Swin-S	0.003	0.002	0.008	0.017	0.146	0.013	0.072	0.021
vs. Swin-B	0.693	0.185	0.101	0.602	0.005	0.009	0.003	0.072

**Table 19:** Statistical significance comparison of BreastSwinFedNetX model on INbreast and CBIS-DDSM using paired *t*-test (α=0.005).

Comparison	INbreast (*p*-Values)				CBIS-DDSM (*p*-Values)			
	Specificity	F1 Score	PR AUC	MCC	Specificity	F1 Score	PR AUC	MCC
vs. Swin-L	0.005	0.012	0.019	0.063	0.592	0.087	0.142	0.203
vs. Swin-T	0.002	0.004	0.048	0.007	0.008	0.006	0.005	0.004
vs. Swin-S	0.003	0.007	0.009	0.025	0.014	0.010	0.019	0.033
vs. Swin-B	0.612	0.198	0.102	0.479	0.017	0.013	0.012	0.006

## Data Availability

The datasets used and/or analyzed during the current study are publicly accessible in BreakHis [48], BUSI [49], INbreast dataset [50], CBIS-DDSM dataset [51], and combine dataset [52].
